# Implications of Solvent Vapor Annealing on Crystallinity and Orientation of Covalent Organic Framework Thin Films

**DOI:** 10.1021/acsomega.5c12800

**Published:** 2026-03-27

**Authors:** Dayanni D. Bhagwandin, Kaushik Chivukula, Evan Wilson, Kirt A. Page, Ly D. Tran, Arthur R. Woll, Hilmar Koerner, Luke A. Baldwin, Tobin J. Marks, Antonio Facchetti, Yu Zhong, Nicholas R. Glavin

**Affiliations:** † Air Force Research Laboratory, Materials and Manufacturing Directorate, WPAFB, Dayton, Ohio 45433, United States; ‡ AV, Inc., Dayton, Ohio 45432, United States; § Department of Materials Science and Engineering, Cornell University, Ithaca, New York 14853, United States; ∥ School of Materials Science and Engineering, Georgia Institute of Technology, Atlanta, Georgia 30332, United States; ⊥ Cornell High Energy Synchrotron Source, Cornell University, Ithaca, New York 14853, United States; # Department of Chemistry and the Materials Research Center, Northwestern University, Evanston, Illinois 60208, United States

## Abstract

Covalent organic frameworks (COFs) are a promising class of crystalline organic polymers that can be processed into many different forms, with a majority existing as insoluble powders and thin films. Still, the optimization of crystallinity and orientation of COF thin films, a precondition for many applications, remains particularly challenging. To address this gap, this study employs grazing-incidence wide-angle X-ray scattering (GIWAXS) to examine solvent vapor annealing (SVA) as a postsynthetic method to enhance the structural ordering of imine-linked COF thin films. The SVA process was first systematically optimized for 1,3,5-tris­(4-aminophenyl)­benzene-*p*-phthalaldehyde (TAPB-PDA) COF thin films through kinetic and thermal studies, with an annealing temperature of 90 °C for 60 min being identified as the optimal treatment condition, which yielded a maximum increase in crystallinity. These optimized conditions were subsequently applied to TAPB-PDA thin films of varying thicknesses and those grown on a diverse range of substrates to evaluate the versatility of the SVA process. These conditions were then utilized for a series of six different COFs with varying chemical compositions and pore sizes. GIWAXS analysis was used to verify the crystalline structure of each COF as well as to obtain a comparative measurement of crystalline volume and orientational order before and after SVA. The SVA process significantly enhances crystallinity in four of the six COFs examined, particularly for those with larger pore sizes (>2.2 nm) and already exhibiting preferential alignment. In particular, 1,3,5-tris­(4-aminophenyl)­benzene-2,5-dihydroxyterephthalaldehyde (TAPB-DHPDA) and TAPB-PDA, both with a pore size of 3.6 nm, demonstrate significant increases in crystalline ordering, with up to a 9-fold increase in intensity and a 61% decrease in full width at half-maximum (FWHM) of the crystalline (100) diffraction peak. The solvent mixture (1,4-dioxane, mesitylene, and 11 M acetic acid) is hypothesized to facilitate this enhancement by enabling imine bond reversibility and creating an environment that promotes crystallite rearrangement. This work demonstrates SVA as an effective method to improve COF thin film quality.

## Graphical abstract

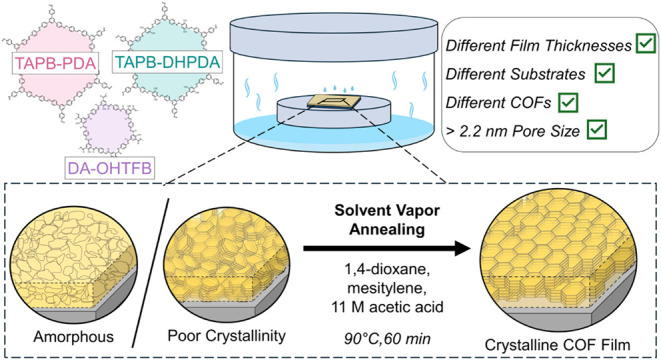

## Introduction

Covalent organic frameworks (COFs) are known for their exceptional thermal stability, low density, and porosity, properties which make them suitable for a variety of thin film applications such as sensing, energy storage, electronics, and optoelectronics.^1,2^ Moreover, COFs can be synthesized from an extensive library of available organic precursors, making their systems highly tunable. Two-dimensional (2D) COFs, in particular, feature an organic structure held together by strong in-plane covalent bonds and weaker out of plane π–π interactions.^3^ Recent work has also highlighted directionally dependent transport properties on oriented 2D COF thin film samples.^4–6^ As many of these applications rely heavily on highly crystalline COF films, the synthesis and processing of well-ordered COF materials as thin films is of utmost importance toward developing advanced microelectronics, sensors, and biocompatible devices.^7,8^ Recently, we demonstrated a solid–liquid growth technique with exceptional potential for fabricating highly crystalline COF thin films with nanometer-scale smoothness directly onto solid substrates.^9,10^ Although this method provides a facile way for producing a variety of COF thin films, optimization of a postsynthetic processing step is necessary to further enhance the crystallinity and orientation of these films.

Postsynthetic processing methods that enhance crystallinity and stabilize COF structures have proven to be important in both powder and thin film systems. In powder samples, steps such as solvent exchange with ultralow surface tension solvents,^11^ drying with supercritical CO_2_ (scCO_2_),^12^ and subjection to continuous flow in a packed-bed reactor^13^ have been utilized to enhance the crystallinity of the product. For COF thin film systems, some works have explored solvent vapor annealing (SVA) as a step to enhance the crystallinity. SVA, which facilitates molecular motion and results in crystallographic rearrangement,^14^ has been used to enhance the crystallinity of organic semiconducting thin films and resulting field effect transistor (FET) mobility.^15^ SVA utilizes a straightforward setup for thin film processing and requires a closed container, solvent, and heating. SVA has been previously utilized for amine-linked TbHz COF and imine-linked LZU1 COF and TpPa COF thin films, where GIWAXS indicated a shift from mixed orientation to parallel alignment.^16^ Additionally, SVA has been shown to improve the crystallinity of TpTG_Cl_ and TpBpy COF thin films, as indicated from powder X-ray diffraction (PXRD), by using a solvent mixture of dioxane, water, and acetic acid.^17^ In this case, the presence of acetic acid and water facilitates the reversibility of the imine bond which helps to reorganize the COF’s backbone. To obtain significant crystallinity for potential device applications and understand the interplay between processing conditions and the resultant COF structure and morphology, it is essential to further investigate SVA as a postsynthetic technique for high-quality COF thin films.

In this work, SVA is performed on imine COF thin films with hexagonal topology and the crystallinity and orientation of these films under various annealing conditions are investigated. First, the SVA procedure is carried out under different annealing times and exposure temperatures in order to determine optimal conditions for improving COF crystallinity and orientation. These optimized conditions were subsequently applied to TAPB-PDA thin films of varying thicknesses and those grown on various substrate to evaluate the versatility of the SVA process. The SVA step is then performed on a variety of different COF samples with differing chemical compositions and pore sizes.

## Results and Discussion

The COF thin films investigated in this study were synthesized using a solid–liquid growth technique, highlighted in prior works,^9^ which uses a two-phase solvent system with methylene chloride and water, and affords a COF thin film directly on the solid substrate (as shown in Figure 1a). Briefly, organic monomers were first dissolved in the bottom organic layer and the substrate was then placed at the bottom. A 1 M solution of acetic acid was then slowly added on top using a syringe to ensure minimal solvent mixing. After 24 h, the films were removed, analyzed, and then subjected to the SVA treatment. More details regarding the thin film synthesis of individual COFs can be found in the Supporting Information. First, a set of TAPB-PDA COF thin films were synthesized by combining amine precursor 1,3,5-tris­(4-aminophenyl)­benzene (TAPB) and an aldehyde precursor *p*-phthalaldehyde (PDA).

**Figure 1:**
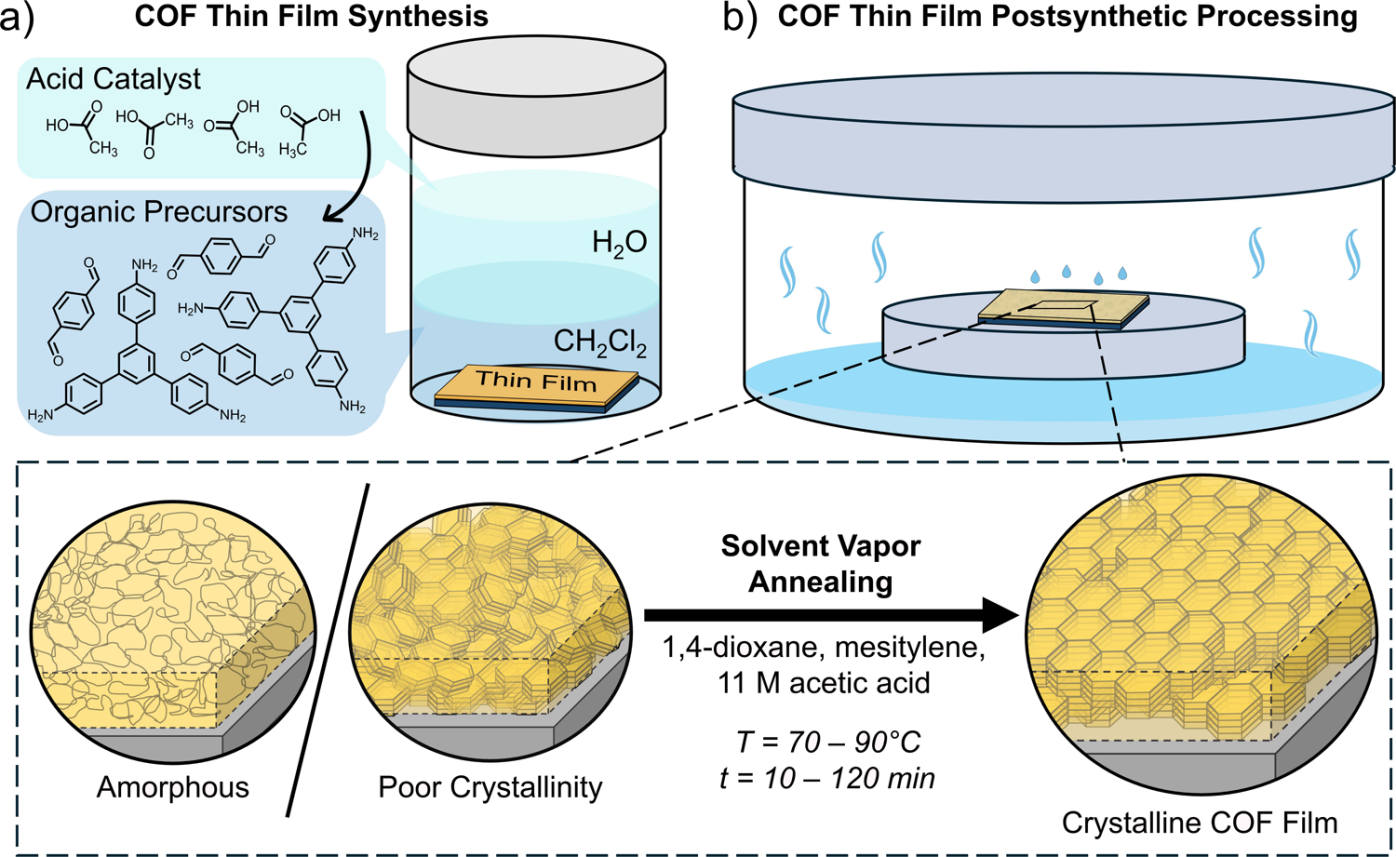
Covalent organic framework (COF) thin film synthesis and postsynthetic processing. (a) Schematic of the interfacial setup for COF thin film synthesis directly on a substrate; (b) schematic of the solvent vapor annealing (SVA) procedure for COF thin films with the experimental conditions used and a schematic of the resulting changes in crystallinity and orientation demonstrated upon solvent annealing.

After thin film growth, samples were removed from the growth beaker, briefly sonicated in acetone, and then analyzed with GIWAXS to determine the as-synthesized crystallinity. Following this, samples were then exposed to SVA using a mixture of 1,4-dioxane, mesitylene, and 10.5 M acetic acid (4:1:1, v/v). This specific solvent mixture has been previously reported to convert amorphous COF powders to crystalline material.^18^ The solution was first added to a wide cylindrical container or jar so that it filled approximately 15% of the volume of the vessel, and a glass Petri dish was placed on top according to the schematic in Figure 1b. The container was then closed, placed on a hot plate, and heated to 70 to 90 °C. Once vapors were formed within the container, the COF thin film was then quickly placed on an elevated surface within the container to prevent submersion in the solution, and then solvent mixture from a separate container at room temperature was pipetted onto the film to fully saturate it. The film was left to stand anywhere from 10 to 120 min and then removed before briefly placing the samples directly on a hot plate set to 90 °C to remove any remaining solvent.

To investigate the effect of SVA annealing time on COF crystallinity, a series of TAPB-PDA COF thin films was subjected to various SVA treatment lengths and the resulting crystallinity was evaluated by grazing-incidence wide-angle X-ray scattering (GIWAXS). The nanometer-scale thickness of the COF films in this study necessitates synchrotron-based GIWAXS for effective characterization. The combination of grazing-incidence geometry with a high intensity, vertically focused X-ray beam permits strong scattering from the COF layer while maximizing signal-to-noise. This provides the sensitivity required to resolve diffraction peaks in these fabricated COF thin films and accurately track their structural enhancements during the SVA process.

All TAPB-PDA films were prepared simultaneously from the same batch in a large beaker with the same concentrations reported for single film growth in the Supporting Information. This batch-growth approach was utilized to mitigate sample-to-sample variability and provide a consistent crystalline starting state across all samples used for the time-dependent annealing study. GIWAXS spectra before SVA treatment shows consistently low intensity peaks for these samples and can be found in the Supporting Information (see Figure S2). GIWAXS spectra for an as-synthesized TAPB-PDA COF sample and SVA-treated TAPB-PDA COF samples (at 70 °C for various times) are shown in Figure 2a. To maintain consistency, the sample was aligned on the stage so that the X-ray footpath contacted the center of each substrate for every GIWAXS measurement. This ensured uniform sampling by measuring the same area on each film before and after treatment. All samples show a peak at 0.2 Å^–1^ corresponding to diffraction of the (100) plane with a *d*-spacing of 3.1 nm and a hexagonal lattice parameter *a* or pore size of 3.6 nm, which closely matches literature values.^9,10,19^ Note that the hexagonal lattice parameter, calculated as $\frac{2}{\surd 3}\times d\;\mathrm{spacing}\;\mathrm{of}\;\left(100\right)\mathrm{Bragg}\;\mathrm{peak}$ , constitutes an upper bound on the physical pore size (see Supporting Information for further details). The (100) diffraction peak observed here is the lowest scattering vector (*q*) peak that is observed in these measurements, with no additional features appearing at lower *q* values within the accessible range of this experimental setup. The anisotropic peak shape indicates that all COF films contain crystallites where the 2D sheets are aligned parallel to the substrate and pore channels are aligned perpendicular. The as-synthesized sample (0 min) has a (100) peak position at the lowest Å^–1^ value, a full width at half-maximum (FWHM) at the highest value, and an intensity at the lowest value in the entire set, suggesting that it has the poorest crystallinity of them all. Peak broadening is an indication of a smaller crystalline domain size, according to the Scherrer equation, while a lower intensity peak indicates a smaller amount of crystalline material throughout.^20^ With regards to the solvent vapor annealing time, 10 to 30 min of annealing show similar changes in peak intensity while 60 min of SVA shows intense diffraction in addition to the appearance of peaks at 0.34 and 0.39 Å^–1^, which correspond to the diffraction of the (110) and (200) planes and distances 1.9 and 1.6 nm, respectively. A horizontal projection (along Q_||_) between 0.05 and 0.07 *Q*_⊥_ (Å ^–1^) was generated from the sample that was annealed for 60 min. Note that this range was chosen to obtain the most peak intensity from the spectra for analysis and all horizontal projections generated in this study averages the same *Q*_⊥_ (Å ^–1^) range, unless otherwise noted (see Figure S3 in the Supporting Information for further details). Sharp diffraction peaks associated with the (100) plane (Figure 2b) and (110) and (200) planes (Figure 2b inset), can be noted in the GIWAXS pattern. The position, intensity, and FWHM of the (100) peak was determined from also taking the horizontal projection (along Q_||_) between between 0.05 and 0.07 *Q*_⊥_ (Å ^–1^) for each sample. The position of the (100) peak across the five samples as a function of annealing time was explored (see Figure 2c). After SVA there is an increase in the Å^–1^ position of the (100) peak. This increase in peak position indicates a slight decrease in the *d*-spacing of the crystalline planes, suggesting a slight increase in the COF layer offset. Meanwhile, the reduction in peak broadening indicates an increased crystallite size.^21^ Additionally, the FWHM and the intensity of the (100) peak is shown in Figure 2c,2d, respectively, as a function of annealing time. While both 60 and 120 min of SVA show the lowest FWHM, which indicates the greatest crystallite size, 60 min also shows the highest increase in intensity, further indicating enhanced crystallinity when compared to all other samples. While the data show a subsequent decrease in peak intensity after 60 min, this observation may be the result of the physical loss of crystalline material from the substrate due to prolonged solvent exposure, which might cause delamination. This hypothesis is supported by initial experimental trials in which prolonged submersion in heated solvent caused most of the COF film to delaminate.

**Figure 2:**
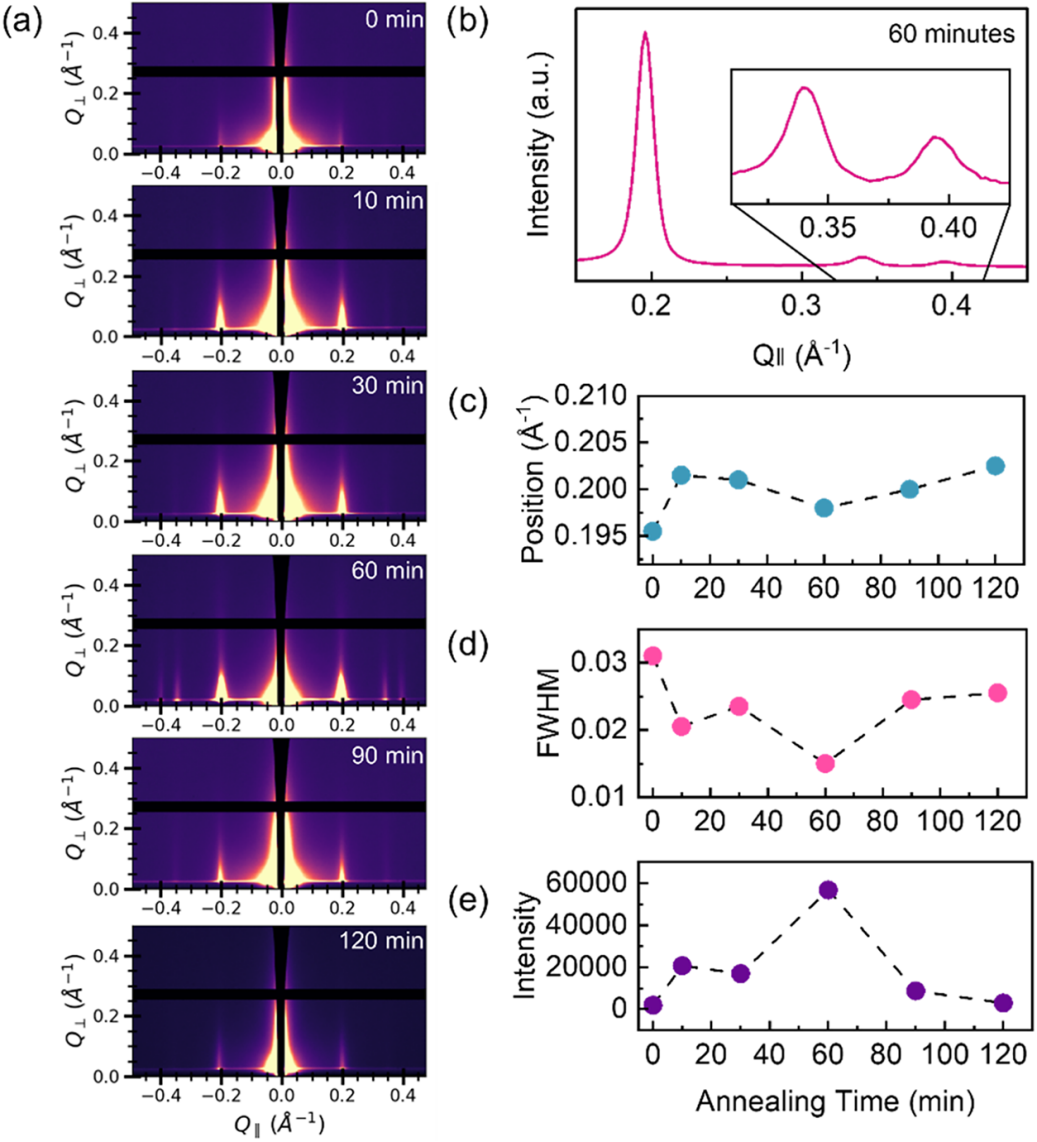
Crystallinity of COF thin films at different solvent annealing times. (a) Grazing-incidence wide-angle X-ray scattering (GIWAXS) of TAPB-PDA COF after solvent annealing for 0, 10, 30, 60, 90, and 120 min (top to bottom); (b) horizontal projection of 60 min sample; (c) plot of (100) peak position of TAPB-PDA COF versus solvent annealing time (min); (d) plot of (100) peak full width at half-maximum (FWHM) of TAPB-PDA COF versus solvent annealing time (min); (e) plot of (100) peak intensity of TAPB-PDA COF versus solvent annealing time (min).

In addition to SVA annealing time, the effect of the annealing temperature on TAPB-PDA COF films was also investigated and the resulting GIWAXS spectra are shown in Figure 3. Note, these samples were prepared from the same batch and then subjected to SVA conditions for 60 min at varying temperatures. Similarly, this batch synthesis protocol was employed to eliminate sample-to-sample variability by ensuring that the initial crystalline state across all thin films was similar (Figure S4). Therefore, this provides a precise comparative measure of structural changes driven purely by the specific temperatures used in the SVA process. The series of GIWAXS images after SVA shows that annealing at 90 °C resulted in the largest increase in crystallinity as indicated by the highest increase in (100) peak intensity (see Figure 3a). The horizontal projection from each of the corresponding samples is also shown in Figure 3b. SVA at 90 °C shows the narrowest and tallest peak with a slight shift to a higher *Q*_||_ value. Based on these studies, solvent annealing at 90 °C for 60 min was chosen as the standard SVA method for TAPB-PDA COF thin films. Temperatures above this were not tested since they exceed the boiling point of dioxane.

**Figure 3:**
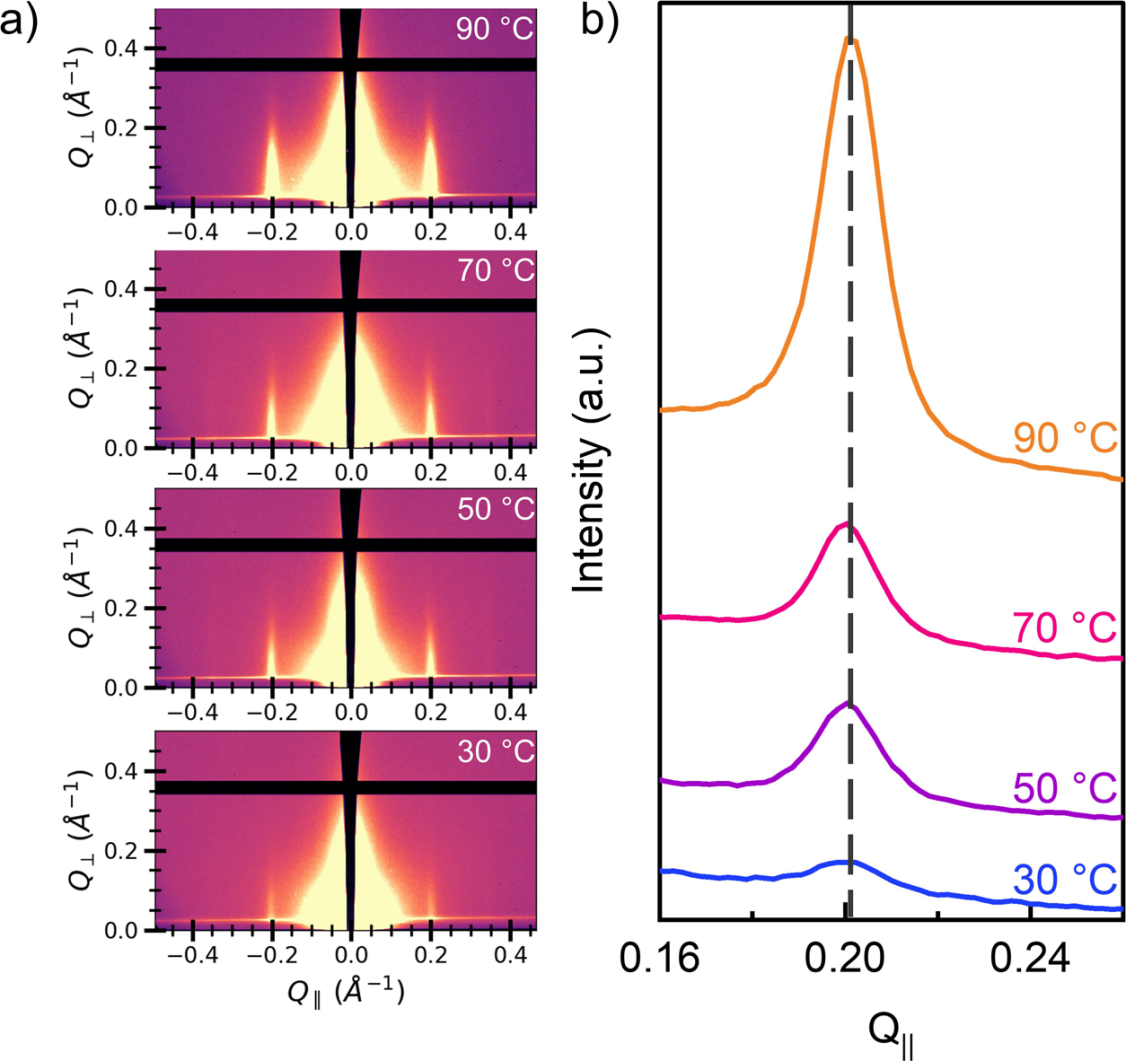
Crystallinity of COF thin films at different solvent annealing temperatures. (a) GIWAXS images of TAPB-PDA COF solvent annealed for 60 min at 30, 50, 70, and 90 °C (bottom to top); (b) horizontal projection of (100) peak of TAPB-PDA COF after solvent annealing for 60 min at 30, 50, 70, and 90 °C (bottom to top).

To evaluate the broader applicability of this optimized SVA treatment beyond the SiO_2_/Si substrate used in initial kinetic and thermal studies, the process was then also performed on TAPB-PDA COF thin films grown on a range of different surfaces. These substrates include glass, indium tin oxide (ITO), silicon, and sapphire, as well as thin film gold- and aluminum- coated silicon. As shown in Figure 4a, GIWAXS analysis was performed both before and after SVA treatment for 60 min at 90 °C to monitor changes in crystallinity. The results are summarized in the peak intensity graph shown in Figure 4b which shows an enhancement in crystallinity for all six substrates tested. Initial intensities (Figure 4b, blue) varied depending on the substrates, with samples containing aluminum and ITO substrates showing poorer preferential alignment compared to the rest of the thin films in the as-synthesized state. The (100) diffraction peak shows an increase in intensity across every sample after the SVA treatment (Figure 4b, pink), with aluminum, silicon, ITO, and sapphire exhibiting the greatest change in (100) peak intensity. This finding confirms that the SVA treatment is a versatile postsynthetic strategy that is effective for improving COF thin film quality regardless of the underlying substrate material, which is a critical feat for integrating these materials into various electronic and sensing device architectures.

GIWAXS analysis of TAPB-PDA COF thin films with varying thicknesses revealed that SVA effectiveness increases with film volume. While a film 10 nm thick remained amorphous after the SVA treatment, thicker samples particularly, one with 100 nm thickness, exhibited the most dramatic enhancement in crystalline ordering, evidenced by a sharp increase in the diffraction intensity coupled with a decrease in FWHM. For GIWAXS images from films with varying thicknesses see Figures S5–S9 in the Supporting Information.

**Figure 4:**
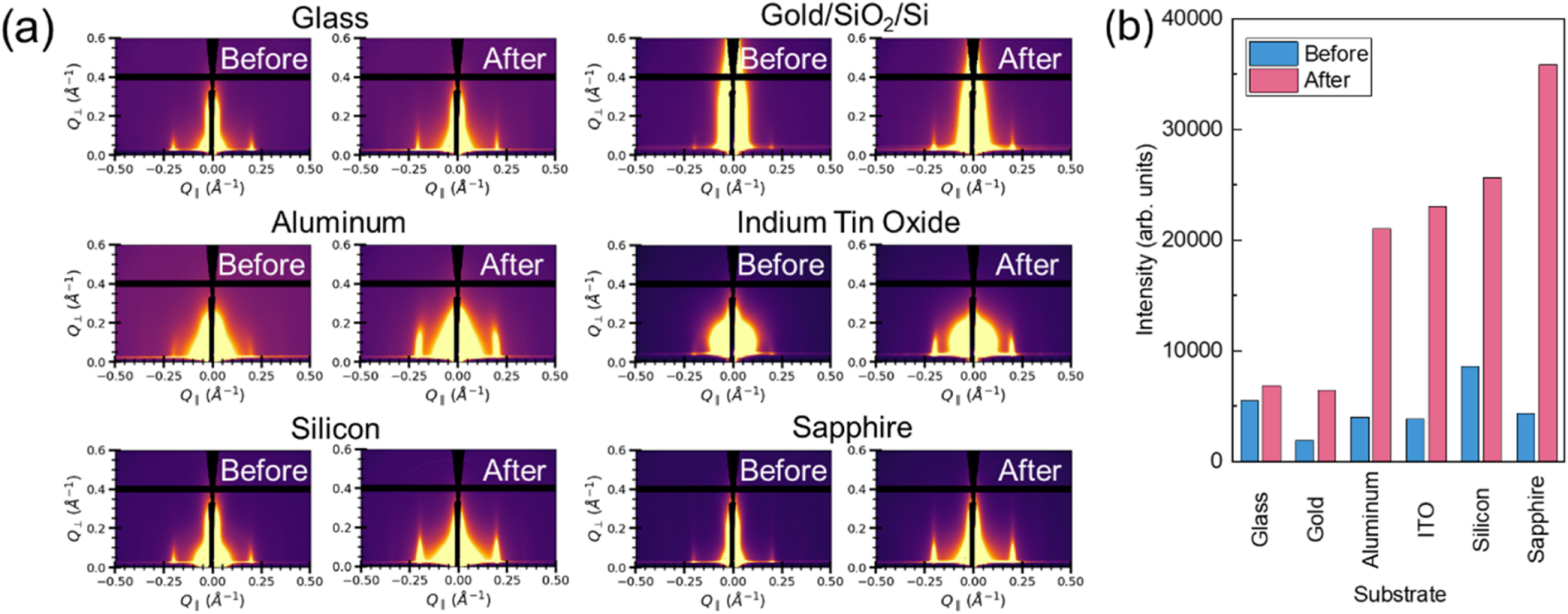
Solvent annealing on different substrates. (a) GIWAXS images of TAPB-PDA COF solvent annealed for 60 min at 90 °C on various substrates (glass, gold, aluminum, indium tin oxide, silicon, and sapphire); (b) graph of intensity of (100) peak from GIWAXS spectra before and after solvent vapor annealing on each substrate.

A series of five additional COF thin films with varying pore sizes and chemistries were also synthesized and subjected to SVA for the optimized conditions of 60 min at 90 °C. Similar to the TAPB-PDA series, the primary diffraction feature for this broader library of COFs was from the (100) plane. Note that no larger-scale periodicities were detected at values below these *q* vectors. TAPA-TFB, TAPB–OHTFB, and TAPB-TFB COF thin films exhibit a mixed orientation, which is shown in Figure 5, with a similar diffraction pattern to powder samples, both prior and post-SVA, where crystallites are oriented randomly throughout the film. This is determined from the isotropic semicircular shape of the peak which indicates negligible preferential crystallite alignment.

**Figure 5:**
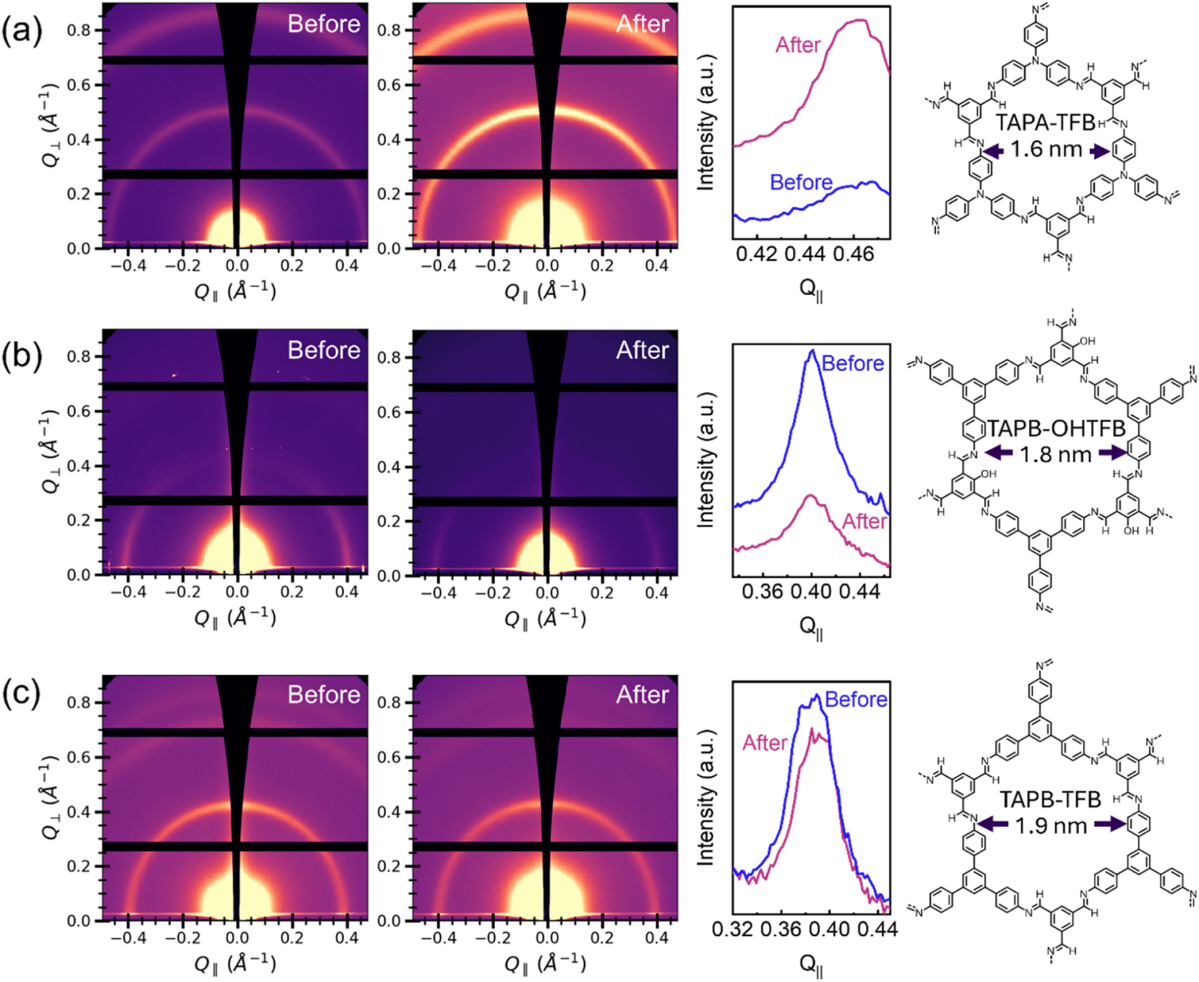
GIWAXS analysis of COFs with mixed orientation. (a) GIWAXS images of TAPA-TFB COF (pore size = 1.6 nm) before and after SVA, horizontal projection of (100) peak; (b) GIWAXS images of TAPB–OHTFB COF (pore size = 1.8 nm) before and after SVA, horizontal projection of (100) peak; (c) GIWAXS images of TAPB-TFB COF (pore size = 1.9 nm) before and after SVA, horizontal projection of (100) peak.

The GIWAXS image recorded before and after the SVA treatment along with the horizontal projection for TAPA-TFB COF, which was synthesized by combining tris­(4-aminophenyl)­amine (TAPA) and 1,3,5-triformylbenzene (TFB) is shown in Figure 5a. The diffraction peaks appear at 0.47 Å^–1^ and 0.85 Å^–1^ indicating *d*-spacings of 1.4 and 0.7 nm and a calculated pore size of 1.6 nm, which is consistent with literature values.^22^ The GIWAXS spectra recorded after SVA show an increase in intensity, indicating improved structural ordering throughout the film. The GIWAXS image and horizontal projection for TAPB-OHTFB COF, which were synthesized with TAPB and 2-hydroxy-1,3,5-triformylbenzene (OHTFB), shows a decrease in intensity after the SVA treatment (see Figure 5b). Nonetheless, the COF (100) peak is detected slightly above 0.40 Å^–1^ both before and after SVA, which indicates a *d*-spacing of 1.6 nm and a pore size of 1.8 nm.^24^ The shape of the peak shows mainly a mixed orientation with some preference for parallel alignment and long range ordering as indicated by the sharpness on the sides of the peak. Similar GIWAXS data for TAPB-TFB COF, which is synthesized by combining TAPB and TFB, show the (100) peak at 0.39 Å^–1^, which indicates a *d*-spacing of 1.6 nm and a pore size of 1.9 nm (see Figure 5c).^23^ Additionally, weak peaks at 0.73 and 0.82 Å^–1^ can be detected, corresponding to the distances 0.9 and 0.8 nm for the (110) and (200) planes, respectively. Here no major differences in terms of intensity are apparent between the GIWAXS images before and after the SVA steps.

The effect of SVA on the crystallographic orientation of highly parallel-oriented COFs was probed and is shown in Figure 6. GIWAXS patterns for these series of samples mainly show a sharp and near-anisotropic (100) diffraction peak in all samples after SVA, which indicates that the (100) plane, or the COF sheets in this case, are aligned parallel to the surface of the substrate.^25^ GIWAXS data before and after the SVA procedure for DA–OHTFB COF, which is synthesized using 1,4-diaminebenzene (DA) and OHTFB, is depicted in Figure 6a. The peak for this COF appears around 0.33 Å^–1^ indicating a *d*-spacing of 1.9 nm and a pore size of 2.2 nm, and matches previous literature values.^6^ While the horizontal projection (along Q_||_) between 0.05 and 0.12 *Q*_⊥_ (Å ^–1^), taken to maximize the range in peak intensity, shows that the intensity of the peak dissipates after the SVA treatment, the GIWAXS peak changes shape from slightly curved to straight (see Figure S14 in the Supporting Information for further detail). Note that a larger range in *Q*_⊥_ (Å ^–1^) was averaged in this sample because of the high degree of crystallinity observed before SVA treatment, compared to the other COF samples. This result indicates that the SVA treatment could affect the orientation of crystallites, causing bond rearrangement so that sheets shift into an entirely parallel orientation. SVA has been shown to regulate the orientation of amine-linked TbHz COF films, when *o*-dichlorobenzene (*o*-DCB) was used as a solvent.^16^ This solvent was suggested to weaken π-π interactions that hold monolayers together and thus provides crystallites with enough mobility to rearrange into a thermodynamically favored product. GIWAXS data for the SVA treatment of TAPB-PDA COF shows substantial improvements in crystallinity (see Figure 6b). Peaks appear in the SVA sample around 0.20 Å^–1^, which indicates a *d*-spacing of 3.1 nm and corresponds to a pore size of 3.6 nm. The horizontal projection for this COF shows that after SVA, there is a large increase in intensity. Finally, the same set of GIWAXS data for TAPB-DHPDA COF, which is synthesized by combining TAPB and 2,5-dihydroxyterephthalaldehyde (DHPDA), is shown in Figure 6c. Sharp diffraction peaks can be noted particularly in the sample that has been treated with SVA. These peaks can also be seen around 0.20 Å^–1^, which indicate a *d*-spacing of 3.2 nm and corresponds to a pore size of 3.6 nm, which is slightly larger than the experimentally determined distances in TAPB-PDA COF.^13^ SVA also greatly effects this sample, as the horizontal projection shows that there is also a large increase in peak height. Therefore, SVA appears to have the strongest effect on the magnitude of crystalline ordering in both TAPB-DHPDA and TAPB-PDA COFs.

To verify that the SVA method does not change the chemical bonding and composition of the film, Raman spectroscopy was employed as shown in Figure 7a. All thin films exhibit no significant change in the spectra measured before and after the SVA treatment for any of the COFs studied. Peaks corresponding to the imine C=N stretching, aromatic ring chain vibrations, and aromatic stretching for C–N and C–C can all be identified in most of the COF samples at 1590 cm^–1^, 1560 cm^–1^, and 1160 cm^–1^, respectively, which closely matches previously reported values.^26^ Additionally, atomic force microscopy (AFM) was used to monitor any morphological changes occurring in the TAPB-PDA COF film (see Figure 7b). TAPB-PDA COF was chosen as a representative case as it shows the most significant change before and after SVA. Thus, after SVA, AFM imaging shows a much smoother film throughout with the appearance of some COF clusters.

**Figure 6:**
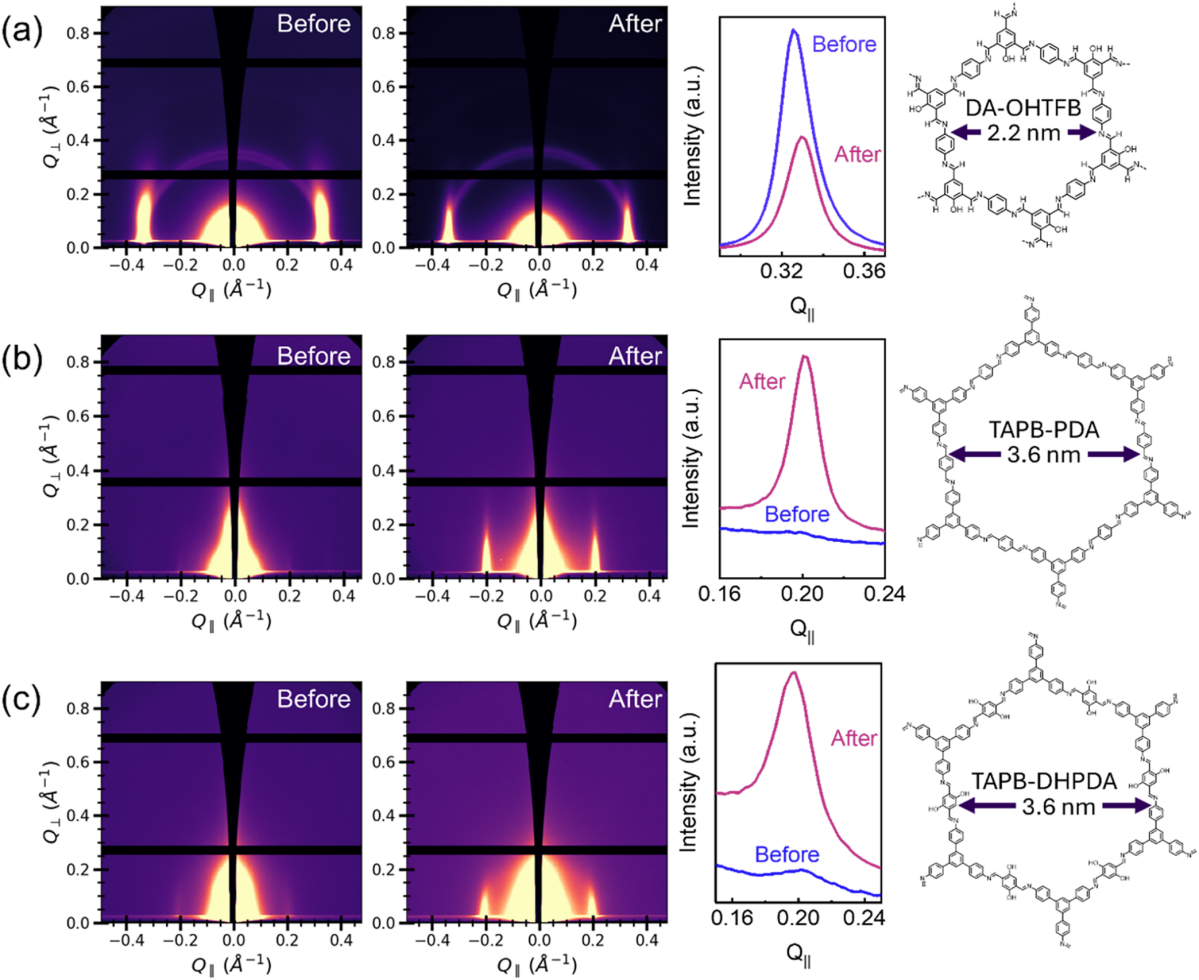
GIWAXS analysis of COFs with parallel orientation. (a) GIWAXS images of DA–OHTFB COF (pore size = 2.2 nm) before and after SVA, horizontal projection of the (100) peak; (b) GIWAXS images of TAPB-PDA COF (pore size = 3.6 nm) before and after SVA, horizontal projection of the (100) peak; (c) GIWAXS images of TAPB-DHPDA COF (pore size = 3.6 nm) before and after SVA, with horizontal projection of (100) peak.

The relationship between SVA and COF film pore size (range of 1.6 to 3.6 nm) was investigated, by monitoring the change in peak position, intensity, and FWHM gathered from the GIWAXS data, as shown in Figure 8. The pore size was calculated for all the COFs used in this study and trends in size were compared against experimental GIWAXS data (see Figure 8a). Most COFs appear to have a minimal change in position of the (100) diffraction peak, suggesting that pore size is not greatly affected by SVA (see Figure 8b). Only TAPB-PDA shows a 5% increase in peak position, suggesting that the pore becomes slightly smaller after SVA. The percentage change in intensity of the (100) peak after SVA was also determined. The results shown in Figure 8c indicate that TAPB-PDA COF has the greatest change with a 9× increase in peak intensity after SVA. Following that, TAPB-DHPDA has the second highest change, with a 5× increase in intensity after SVA. The percent change in FWHM of the (100) peak for all COFs is shown in Figure 8d. Results indicate that TAPB-PDA COF and TAPB-DHPDA COF have the greatest negative change in FWHM, with a 61% and 37% decrease, respectively, corresponding to an increase in crystallite size. Both TAPB-PDA and TAPB-DHPDA start as a more amorphous film and then can be subsequently crystallized through the SVA process postsynthetically.

**Figure 7:**
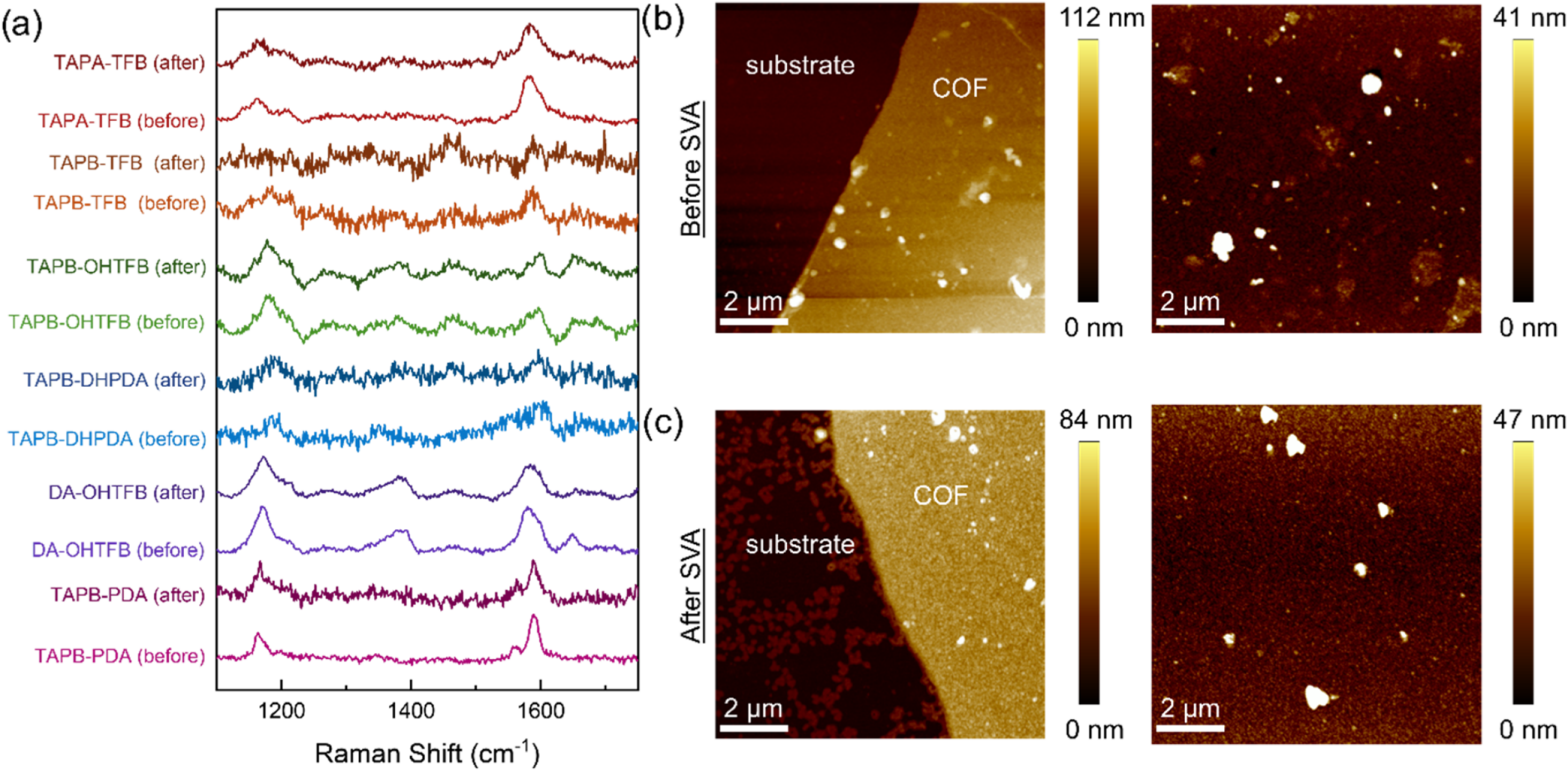
Raman and AFM characterization before and after SVA. (a) Raman spectra of the indicated COF films before and after SVA; (b) Atomic Force Microscopy (AFM) images of a TAPB-PDA COF film before; (c) and after SVA.

Overall, our data suggest that SVA has a greater effect on COFs with larger pore sizes (≥2.2 nm) and initial lower crystallinity. While DA–OHTFB COFs (pore size = 2.2 nm) show a change in preferred orientation compared to the rest, as indicated from the change in the (100) diffraction peak shape, both TAPB-PDA (pore size = 3.6 nm) and TAPB-DHPDA (pore size = 3.6 nm) exhibit significant changes in magnitude of crystalline ordering throughout the film. This may be due to differences in backbone rigidity across the series, which in turn modifies the polymer’s ability to reorganize and crystallize.^27^ SVA utilizing acetic acid has been shown to improve the crystallinity of COF thin films due to the presence of the acid catalyst which facilitates the bond reversibility and rearranges the crystallites into a more organized structure.^17,28^ Additionally, the combination of mesitylene, water, and 1,4-dioxane has been shown to play an important role in which dioxane acts as a bridging solvent between the hydrophobic and hydrophilic solvents creating an emulsion for the COF materials.^29^ The COFs with a pore size smaller than 2.2 nm did not show any significant change in orientation or ordering after SVA. This also suggests that the effects of SVA may be dependent on orientation, making it difficult for solvent vapors to penetrate a film comprised of randomly oriented crystallites of smaller pore sizes. Moreso, other works have fine-tuned COF pore sizes to selectively control capabilities such as sieving, porosity, filtering and adsorption.^30–32^ It is therefore likely that the larger pore size is less selective for solvent permeation simply based on the available surface area.

The effects observed in this study also indicate that the resulting crystallinity through SVA might be dependent on the starting crystallinity of the film. In this case, films that started as more amorphous, showed the greatest increase in crystallinity, compared to films that already had a greater degree of crystallinity. Similarly, other works have pointed to obtaining COF material with substantially improved crystallinity from an amorphous polymeric network that is first formed.^18^ It is also noted that the three COFs with mixed orientation and less significant changes from SVA (TAPA-TFB, TAPB–OHTFB, and TAPB-TFB COFs) are comprised of only tritopic C_3_-symmetric monomers, giving rise to a 3 + 3 topology and only six imine bonds per pore. The other three COFs with parallel orientation and a greater observed change from SVA (DA–OHTFB, TAPB-PDA, and TAPB-DHPDA COFs) are synthesized with tritopic amines and ditopic C_2_-symmetric aldehydes, giving rise to a 3 + 2 topology and 12 imine bonds per pore. The higher density of imine bonds per pore could offer less structural rigidity through a greater chance of reorganization from the SVA conditions. This disparity in SVA efficacy based on pore size and topology is likely linked to the stiffness of the organic network. COFs with a smaller pore size and 3 + 3 topology possess a higher degree of backbone rigidity and steric hindrance, which may limit the molecular mobility required for significant structural reorganization. On the other hand, COFs with a larger pore size and 3 + 2 topology appear more flexible due to the conformational freedom from the network. Overall, SVA might be most suitable as a technique to enhance the crystallinity in films where the COF is inherently fragile or prone to collapse.^11,33^

## Conclusions

In this work, we demonstrate that the postsynthetic SVA step is a powerful technique for enhancing the structural ordering and crystallinity of several imine-linked covalent organic framework thin films. In TAPB-PDA COF thin films, solvent vapor annealing at 90 °C for 60 min was identified as the optimal treatment condition. Additionally, SVA also enhances crystallinity across varying film thicknesses and on a diverse range of substrates including sapphire, silicon, and indium tin oxide. Utilizing the same procedure for a series of six different COF films revealed differing results between the effectiveness of SVA and the inherent pore size, crystallinity, and crystallite orientation of the film. GIWAXS data confirmed that SVA significantly improves the crystallinity in frameworks with larger pore sizes (≥2.2 nm) and films that exhibit some preferential crystallite alignment and amorphous-like film morphology. The greatest improvement was observed in TAPB-PDA and TAPB-DHPDA COFs, where TAPB-PDA COF exhibited up to a 9-fold increase in peak intensity and more than a 60% decrease in FWHM. These results are likely due to the SVA process facilitating the rearrangement of a more flexible or initially disorganized structure. Conversely, COF films with a smaller pore size and isotropic crystallite orientation showed minimal change in crystallinity, suggesting that these structural factors might limit solvent penetration and structural reorganization. This work not only establishes SVA as a facile and effective postsynthetic strategy for improving the quality of COF thin films but also provides insight into the interplay between pore size, crystallite orientation, and solvent-induced mobility in COF thin film systems. By optimizing this postsynthetic process, better control of COF thin films’ crystallinity and ordering can be obtained, thus facilitating their use in advanced microelectronics, sensors, and other functional devices.

**Figure 8:**
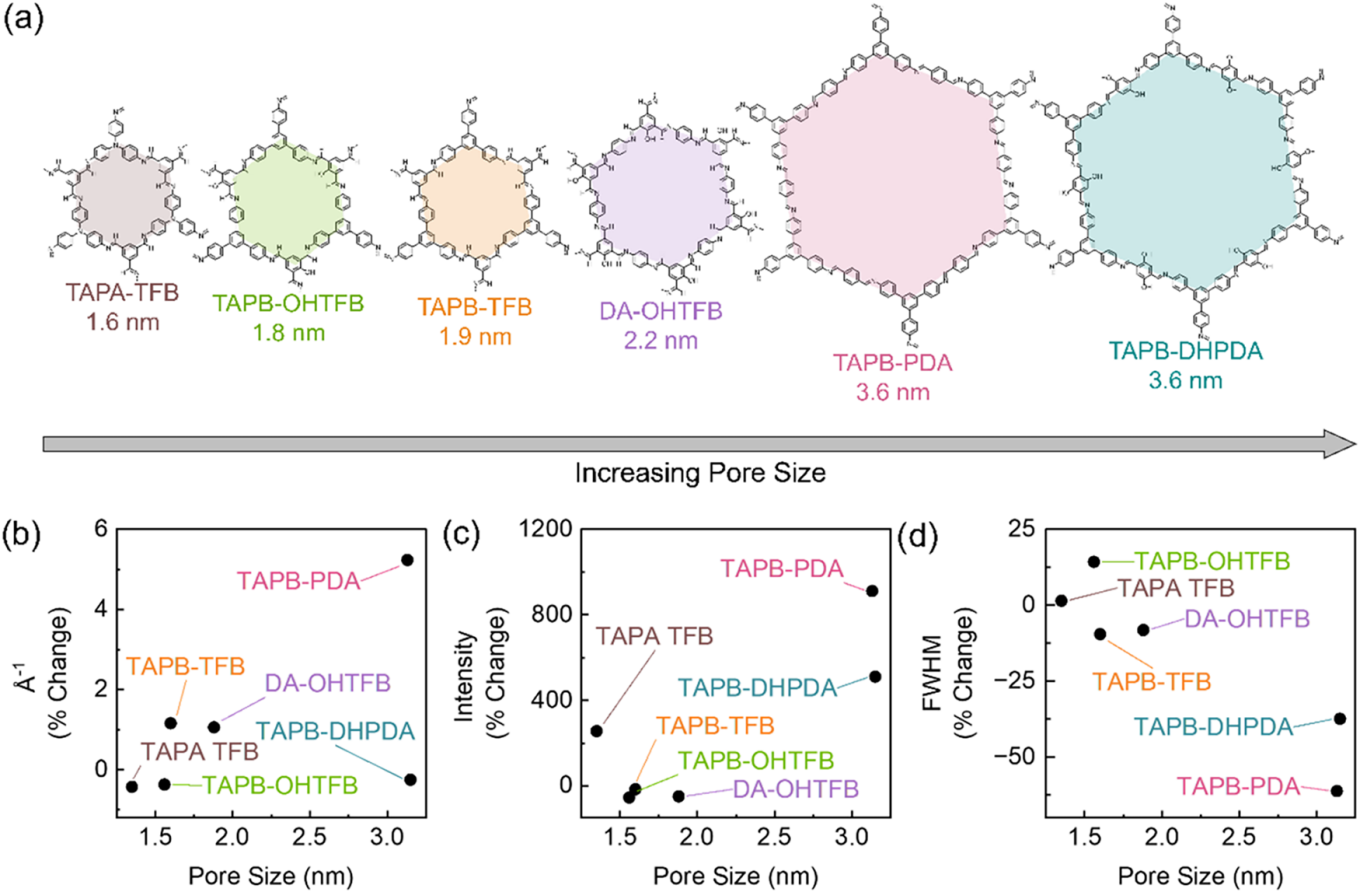
Influence of solvent annealing of six COF thin films with different pore sizes. (a) Schematic representations of COFs in order of increasing pore size: TAPA-TFB COF (brown), TAPB–OHTFB COF (green), TAPB-TFB COF (orange), DA–OHTFB COF (purple), TAPB-PDA COF (pink), TAPB-DHPDA COF (blue); (b) percent change in position of (100) peak after SVA; (c) percent change in intensity of the (100) diffraction peak after SVA; (d) percent change in full width at half-maximum (FWHM) of the (100) diffraction peak after SVA.

## Methods

### Reagents

All reagents were purchased from commercial suppliers. 1,3,5-tris­(4-aminophenyl)­benzene (TAPB) was purchased from Ambeed, Inc. with a 97% purity. and p-phthalaldehyde (PDA) was purchased from Aldrich with a 99% purity. Tris­(4-aminophenyl)­amine (TAPA) was purchased from ChemScene with a 96% purity. 1,3,5-triformylbenzene (TFB) was purchased from Ambeed, Inc. with a 96% purity. 2-hydroxy-1,3,5-triformylbenzene (OHTFB) was purchased from Combi-Blocks with a 97% purity. Benzene-1,4-diamine (DA) was purchased from Combi-Blocks with a 97% purity. 2,5-dihydroxyterephthalaldehyde (DHPDA) was purchased from Ambeed, Inc. with a 95% purity.

### Data

All data were plotted using Origin Pro.

### Grazing-Incidence Wide-Angle X-ray Scattering (GIWAXS)

The grazing-incidence X-ray scattering measurements were carried out at the Functional Materials Beamline (FMB) of the Materials Solutions Network at the Cornell High Energy Synchrotron Source (MSN– CHESS). An X-ray beam energy of 9.7 keV (λ = 1.28 Å) was selected using the 111 reflection of a single-bounce, HPHT diamond monochromator.^34^ Harmonic rejection and vertical focusing are provided by a 1 m long, bendable, rhodium-coated monochromatic mirror located approximately 7 m upstream of the experimental hutch at an incident angle of 4 milliradians. Experiments were carried out in “bulk-beam” mode and the monochromatic mirror was used to focus the beam into a spot approximately (0.045 × 0.5) mm^2^ at the sample position, with a total flux of approximately 10^12^ photons/s at 100 mA beam current.^35^ The samples were mounted on a 4-axis goniometer. Further details of the sample alignment procedure and determination of the critical angle can be found in the Supporting Information. Experiments were performed over a range of incident angles, both below and above the film critical angle. Scattering images were collected on a Pilatus 300 K detector (Dectris, Baden, Switzerland) with a sample-to-detector distance of 42–43 cm. Detector images were calibrated using silver behenate and employing the calibration utility provided in the pyFAI software package^36^ to convert the images to *q*-space. Under this geometric configuration, the highest accessible scattering vector for *q* values in the in-plane direction was approximately 0.55 Å^–1^ and out-of-plane direction was approximately 1.1 Å^–1^. In-house code was developed to correct and analyze the scattering images in order to produce intensity versus scattering vector, *q*(Å^–1^), plots.

### Raman Spectroscopy

Raman spectra were collected using 633 nm excitation wavelength in a Renishaw in Via Raman microscope with an 1800 lines/mm grating. A 50× objective lens was used to focus the incident laser beam on the samples, which were irradiated by an effective laser power of ∼0.3 mW. Raman spectra were collected from the samples with 30 s acquisition times. One acquisition was collected from five different regions on a given film, and the average value was taken at each point.

### Atomic Force Microscopy

AFM images were collected using a Bruker Dimension Icon with Scan Asyst in noncontact tapping mode. The images were quantitatively analyzed for roughness and thickness using NanoScope Analysis.

## Supplementary Material



## Notes

Covalent organic framework (COF) thin film synthesis, photos of COF films, verification of film uniformity, all GIWAXS images before and after solvent vapor annealing (SVA), GIWAXS images with *Q*_⊥_ (Å ^–1^) range used for horizontal projections, GIWAXS images with films of different thicknesses, tables with values for GIWAXS analysis, calculation for determining pore size, determination of the critical angle, and beam footprint analysis (PDF)
