# Supplementary material for: Implications of Solvent Vapor Annealing on Crystallinity and Orientation of Covalent Organic Framework Thin Films
Source: ACS Omega. 2026 Mar 27;11(13):20795–805. doi: 10.1021/acsomega.5c12800 (PMC13063097; doi:10.1021/acsomega.5c12800)
Supplement: Supplementary file 1 [file ao5c12800_si_001.pdf]

Supporting Information

Implications of Solvent Vapor Annealing on Crystallinity and Orientation of Covalent Organic Framework Thin Films

Dayanni D. Bhagwandin [a,b], Kaushik Chivukula[c], Evan Wilson[d], Kirt A. Page[a,b,e], Ly D. Tran[a], Arthur R. Woll[e], Hilmar Koerner[a], Luke A. Baldwin[a], Tobin J. Marks[f], Antonio Facchetti[d,f], Yu Zhong[c], Nicholas R. Glavin[a]*

[a]*Air Force Research Laboratory, Materials and Manufacturing Directorate, WPAFB, OH 45433 USA*

[b]*AV, Inc., Dayton, OH 45432 USA*

[c]*Department of Materials Science and Engineering, Cornell University, Ithaca, NY 14853, USA*

[d]*School of Materials Science and Engineering, Georgia Institute of Technology Atlanta, GA 30332, USA*

[e]*Cornell High Energy Synchrotron Source, Cornell University, Ithaca, New York 14853, USA*

[f]*Department of Chemistry and the Materials Research Center, Northwestern University, Evanston, IL, 60208, USA*

Table of Contents

COF Thin Film Synthesis S3

Photo of TAPB-PDA COF Film S4

Verification of Film Uniformity S5

GIWAXS Analysis of TAPB-PDA COF Film S5

TAPB-PDA COF Films with Different Thicknesses S8

Photos of TAPA-TFB COF, TAPB-OHTFB COF, TAPB-TFB COF, and DA-OHTFB COF Films S12

GIWAXS Analysis of DA-OHTFB COF Film S13

Photo of TAPB-OHPDA COF Film S15

GIWAXS Data Analysis S16

Determination of COF Pore Size from GIWAXS S18

Determination of the Critical Angle S19

Beam Footprint Analysis S20

References S21

COF Thin Film Synthesis

Synthesis of TAPB-PDA COF Thin Film

1,3,5-tris(4-aminophenyl)benzene (TAPB) (13 mg, 0.04 mmol, 1.0 eq) and *p*-phthalaldehyde (PDA) (7.5 mg, 0.06 mmol, 1.5 eq) were added to a 20 mL scintillation vial and mixed with methylene chloride (8 mL). The mixture was then sonicated in the closed vial for 5 minutes to ensure the precursors were fully dissolved. Then an SiO_2/Si substrate was placed at the bottom of the vial. Following this, 8 mL of aqueous 1 M acetic acid was slowly syringed on top of the organic layer to ensure minimal solvent mixing. The setup was left to stand for 24 hours. Once the film was removed, it was briefly sonicated to remove any large COF residue.

Synthesis of TAPA-TFB COF Thin Film

Tris(4-aminophenyl)amine (TAPA) (11 mg, 0.04 mmol, 1.0 eq) and 1,3,5-triformylbenzene (TFB) (6.1 mg, 0.04 mmol, 1.0 eq) were added to a 20 mL scintillation vial and mixed with methylene chloride (8 mL). The mixture was then sonicated in the closed vial for 5 minutes to ensure the precursors were fully dissolved. Then an SiO_2/Si substrate was placed at the bottom of the vial. Following this, 8 mL of aqueous 1 M acetic acid was slowly syringed on top of the organic layer to ensure minimal solvent mixing. The setup was left to stand for 24 hours. Once the film was removed, it was briefly sonicated to remove any large COF residue.

Synthesis of TAPB-OHTFB COF Thin Film

1,3,5-tris(4-aminophenyl)benzene (TAPB) (13 mg, 0.04 mmol, 1.0 eq) and 2-hydroxy-1,3,5-triformylbenzene (OHTFB) (6.7 mg, 0.04 mmol, 1.0 eq) were added to a 20 mL scintillation vial and mixed with methylene chloride (8 mL). The mixture was then sonicated in the closed vial for 5 minutes to ensure the precursors were fully dissolved. Then an SiO_2/Si substrate was placed at the bottom of the vial. Following this, 8 mL of aqueous 1 M acetic acid was slowly syringed on top of the organic layer to ensure minimal solvent mixing. The setup was left to stand for 24 hours. Once the film was removed, it was briefly sonicated to remove any large COF residue.

Synthesis of TAPB-TFB COF Thin Film

1,3,5-tris(4-aminophenyl)benzene (TAPB) (13 mg, 0.04 mmol, 1.0 eq) and 1,3,5-triformylbenzene (TFB) (6.1 mg, 0.04 mmol, 1.0 eq) were added to a 20 mL scintillation vial and mixed with methylene chloride (8 mL). The mixture was then sonicated in the closed vial for 5 minutes to ensure the precursors were fully dissolved. Then an SiO_2/Si substrate was placed at the bottom of the vial. Following this, 8 mL of aqueous 1 M acetic acid was slowly syringed on top of the organic layer to ensure minimal solvent

mixing. The setup was left to stand for 24 hours. Once the film was removed, it was briefly sonicated to remove any large COF residue.

Synthesis of DA-OHTFB COF Thin Film

Benzene-1,4-diamine (DA) (6.1 mg, 0.06 mmol, 1.0 eq) and 2-hydroxy-1,3,5-triformylbenzene (OHTFB) (6.7 mg, 0.04 mmol, 0.7 eq) were added to a 20 mL scintillation vial and mixed with methylene chloride (8 mL). The mixture was then sonicated in the closed vial for 5 minutes to ensure the precursors were fully dissolved. Then an SiO_2/Si substrate was placed at the bottom of the vial. Following this, 8 mL of aqueous 1 M acetic acid was slowly syringed on top of the organic layer to ensure minimal solvent mixing. The setup was left to stand for 24 hours. Once the film was removed, it was briefly sonicated to remove any large COF residue.

Synthesis of TAPB-DHPDA COF Thin Film

1,3,5-tris(4-aminophenyl)benzene (TAPB) (13 mg, 0.04 mmol, 1.0 eq) and 2,5-dihydroxyterephthalaldehyde (DHPDA) (9.4 mg, 0.06 mmol, 1.5 eq) were added to a 20 mL scintillation vial and mixed with methylene chloride (8 mL). The mixture was then sonicated in the closed vial for 5 minutes to ensure the precursors were fully dissolved. Then an SiO_2/Si substrate was placed at the bottom of the vial. Following this, 8 mL of aqueous 1 M acetic acid was slowly syringed on top of the organic layer to ensure minimal solvent mixing. The setup was left to stand for 24 hours. Once the film was removed, it was briefly sonicated to remove any large COF residue.

Photo of TAPB-PDA COF Film

Note that the substrates are approximately 1.2 x 2.0 cm in size.

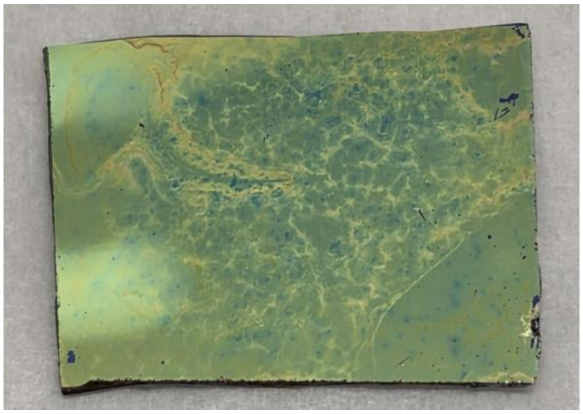

Figure S1. Photo of TAPB-PDA COF on SiO_2/Si substrate

Verification of Film Uniformity

To confirm the spatial uniformity of the COF thin films and the reproducibility of the GIWAXS data, representative samples were first scanned in the incidence angle to find the grazing condition. A scan in z was then performed to ensure optimal positioning. The same alignment was kept while moving to a different position in sam x on the sample surface. Scans were repeated to ensure no real variation in the scattering occurred at different positions on the sample.

GIWAXS Analysis of TAPB-PDA COF Film

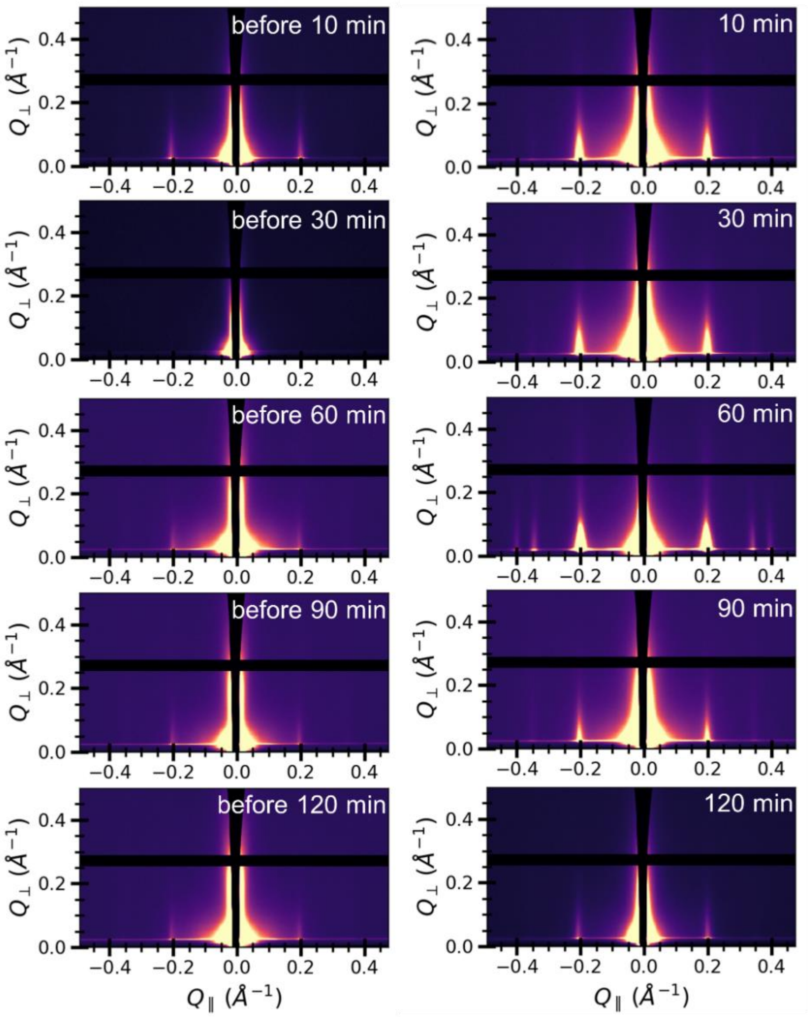

Figure S2. GIWAXS spectra of samples before and after SVA treatment at different times (seen in **Figure 2**).

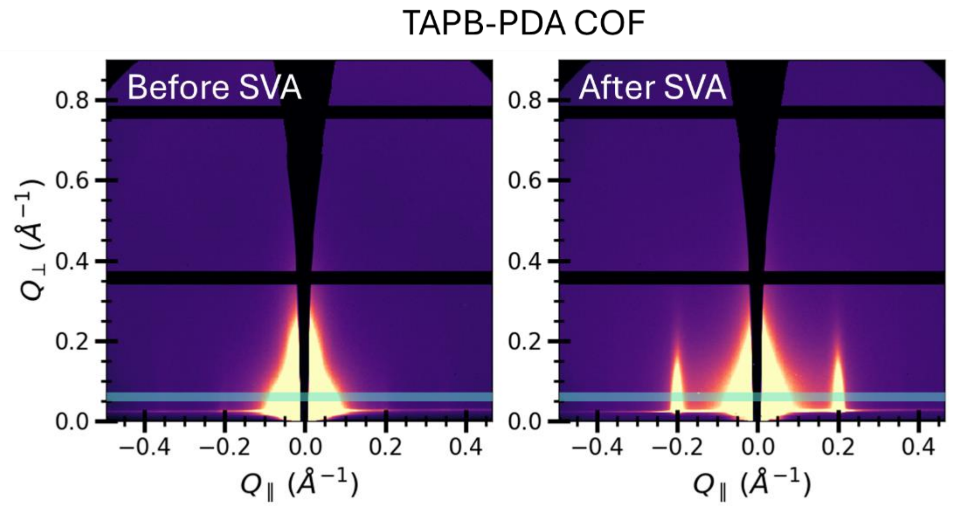

Figure S3. 0.05 to 0.07 Q⊥ (Å $^{-1}$) range for azimuthal average

The above image shows the Q⊥ (Å $^{-1}$) range used to take the azimuthal average and generate 1-D plots for all COFs in this study (except for DA-OHTFB COF).

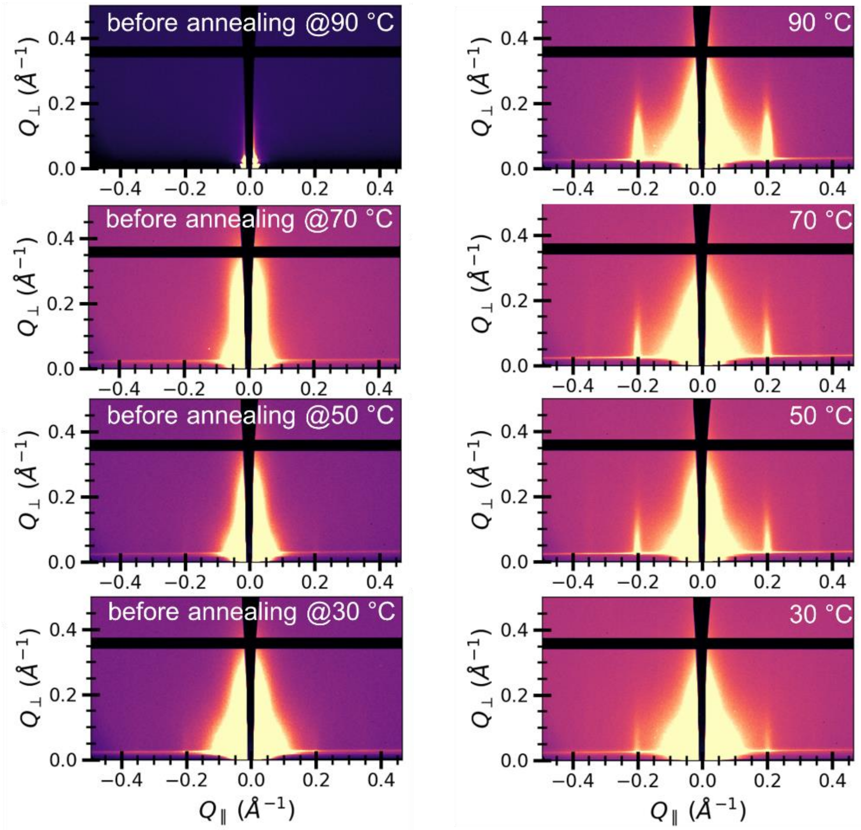

Figure S4. GIWAXS spectra of samples before and after SVA treatment at different temperatures (seen in **Figure 3**)

TAPB-PDA COF Films with Different Thicknesses

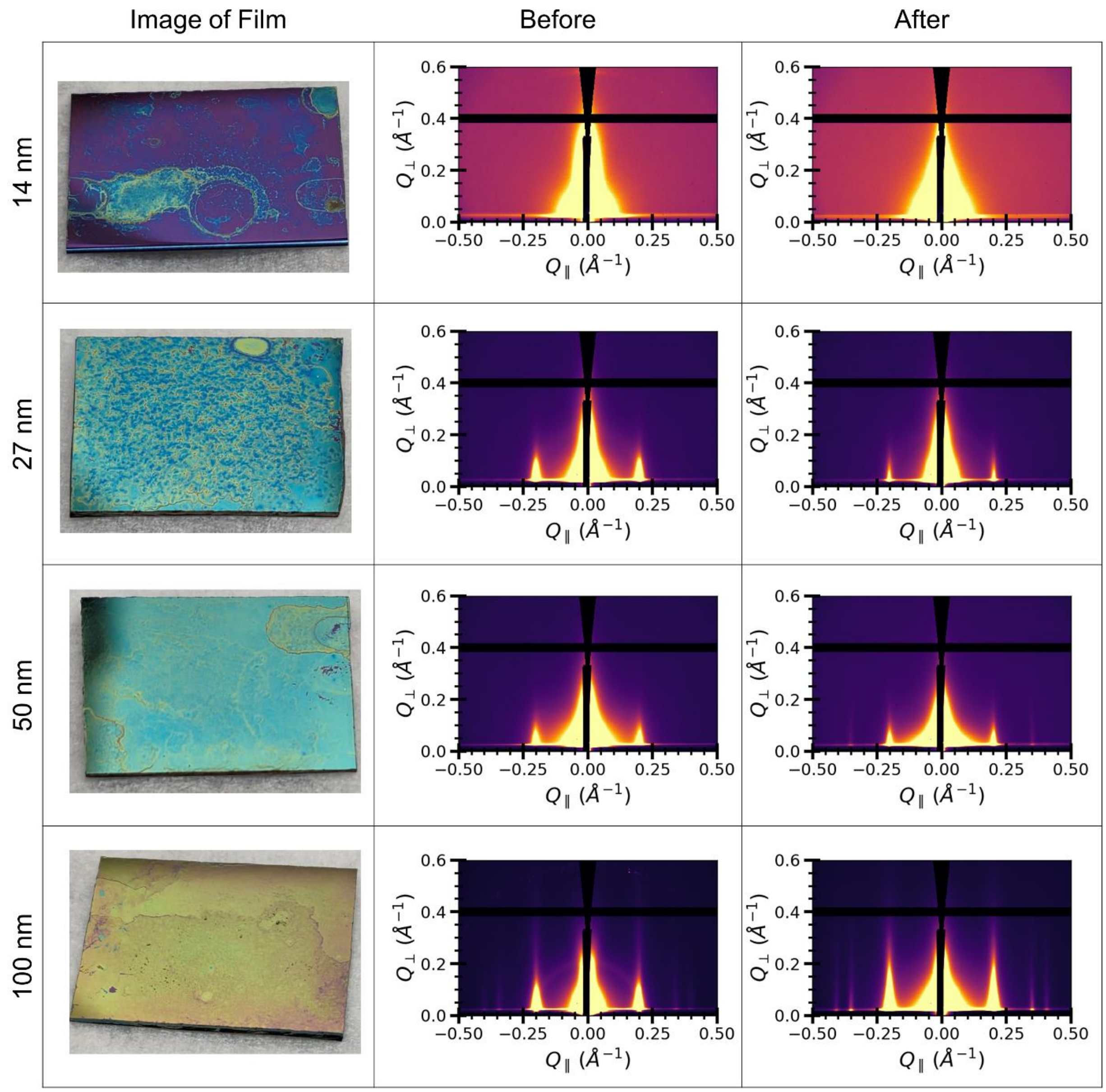

Figure S5. GIWAXS spectra of TAPB-PDA COF films with various thicknesses on SiO_2/Si

To afford TAPB-PDA COF thin films with different thicknesses, the standard protocol for the synthesis of TAPB-PDA COF thin films from above was followed but for different times. The 14 nm and 27 nm sample were afforded by changing the growth times to 8

and 16 hours respectively. Note that times less than 8 hours produced little to no film on the substrate. The film that is 50 nm thick was afforded by following the regular 24-hour growth procedure. Finally, the film that was 100 nm thick was afforded by performing two consecutive 24-hour growth cycles on the same substrate.

GIWAXS analysis was performed on all samples before and after SVA treatment for 60 minutes at 90°C. Results are depicted in **Figure S5**. The thinnest sample of 14 nm shows no indication of crystallinity before and after SVA treatment. This could be due to the fact that it is under the threshold of detection or growth time does not allow for enough COF crystallization to initially take place, making it difficult for the SVA treatment to enhance a system that is totally disordered. The sample that is 27 nm thick shows a decrease in the full-width half-max of the (100) peak after SVA, though there is a loss in intensity. The sample that is 50 nm thick also shows a decrease in FWHM of the (100) peak and the appearance of peaks related to the diffraction of the (110) and (200) planes. The sample that is 100 nm thick shows good crystallinity before but also shows a sharp increase in the intensity of all diffraction peaks as well as a decrease in the FWHM of the (100) peak. Overall this data shows that the SVA is more effective at increasing the crystalline volume of thicker samples, and samples 10 nm in thickness or below made using this procedure cannot be made crystalline with SVA.

Note that the height analysis and AFM images was performed before the SVA treatment took place.

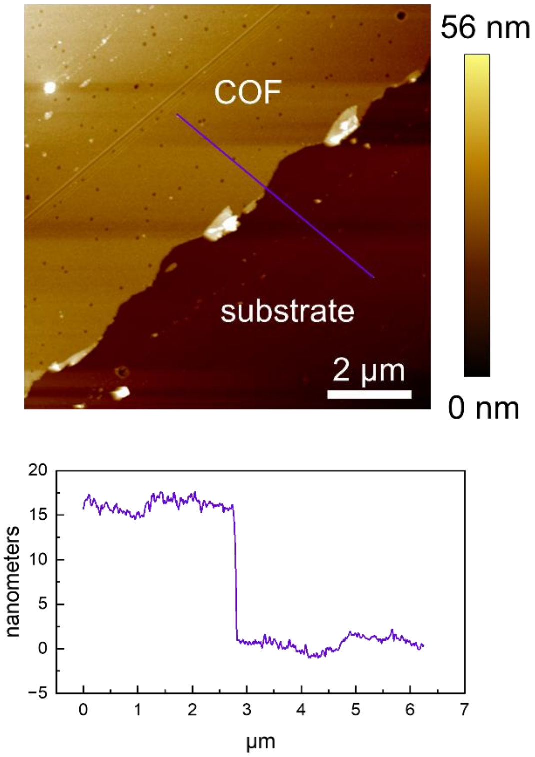

Figure S6. 14 nm thick TAPB-PDA COF film

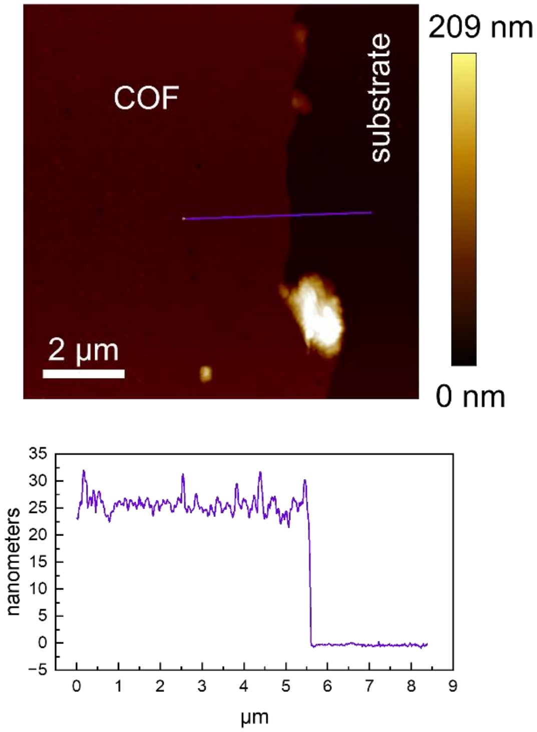

Figure S7. 27 nm thick TAPB-PDA COF film

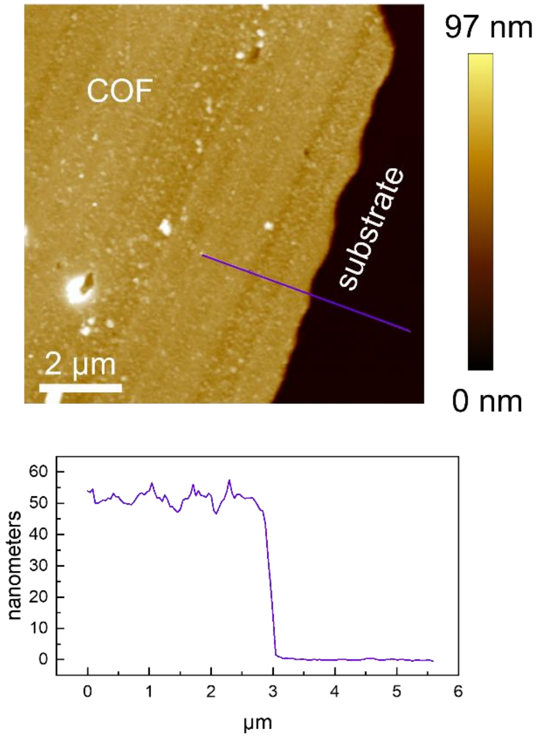

Figure S8. 50 nm thick TAPB-PDA COF film

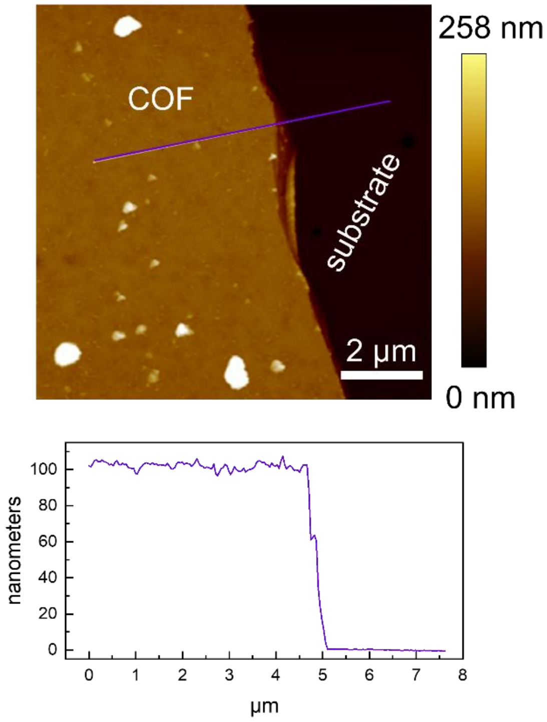

Figure S9. 100 nm thick TAPB-PDA COF film

Photos of TAPA-TFB COF, TAPB-OHTFB COF, TAPB-TFB COF, and DA-OHTFB COF Films

The varied iridescent patterns observed macroscopically are primarily attributed to slight thin-film thickness gradients across the sample.

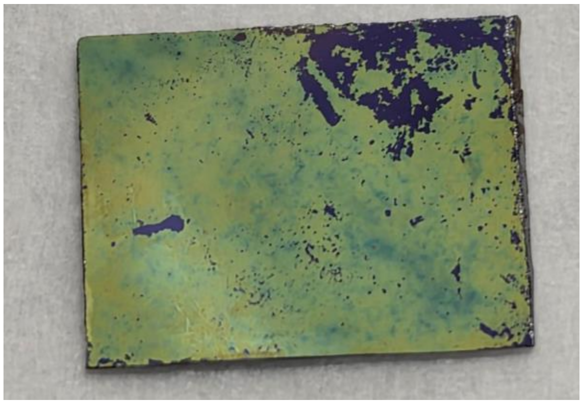

Figure S10. Photo of TAPA-TFB COF on SiO_2/Si substrate

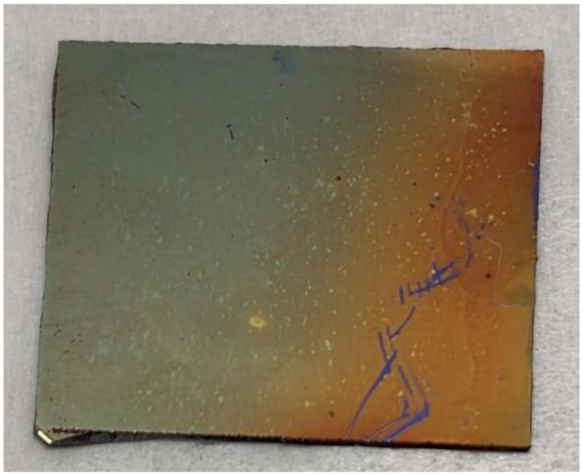

Figure S11. Photo of TAPB-OHTFB on SiO_2/Si substrate

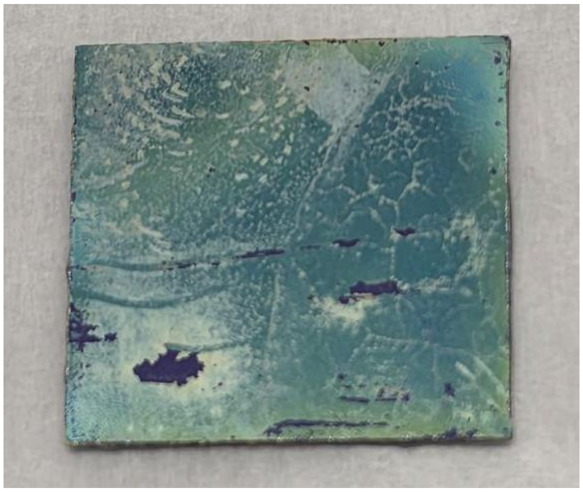

Figure S12. Photo of TAPB-TFB COF on SiO_2/Si substrate

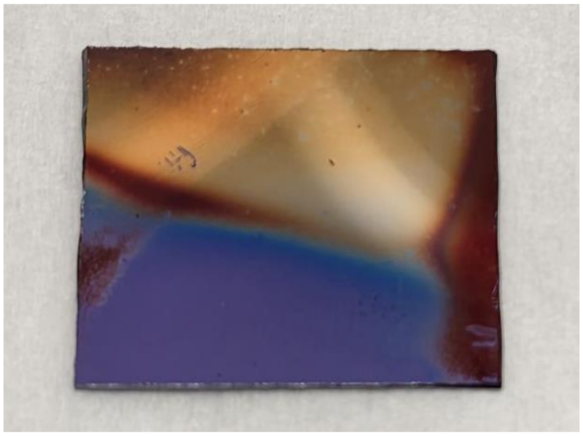

Figure S13. Photo of DA-OHTFB COF (orange yellow) on SiO_2/Si substrate (purple)

GIWAXS Analysis of DA-OHTFB COF Film

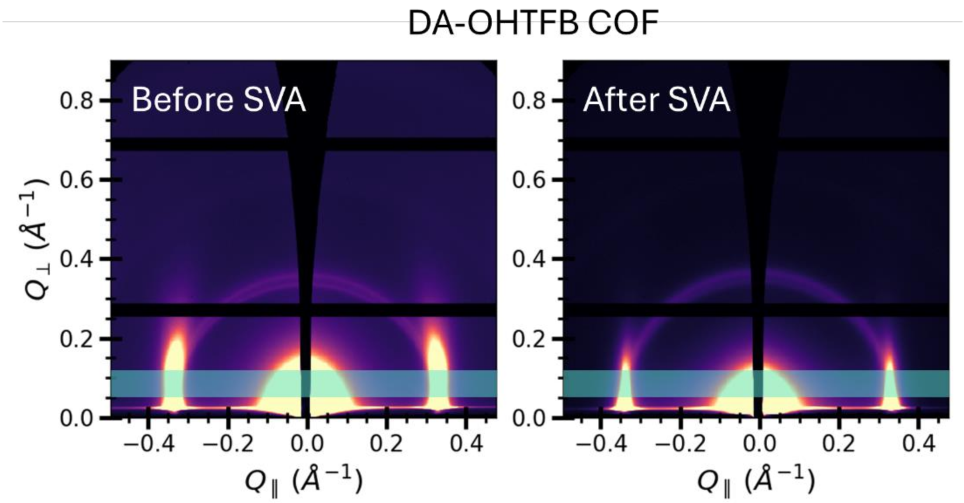

Figure S14. 0.05 to 0.12 Q⊥ ($Å^{-1}$) range for azimuthal average

The above image shows the Q⊥ ($Å^{-1}$) range used to take the azimuthal average and generate the 1-D plot and (100) peak data specifically for DA-OHTFB COF.

Photo of TAPB-DHPDA COF Film

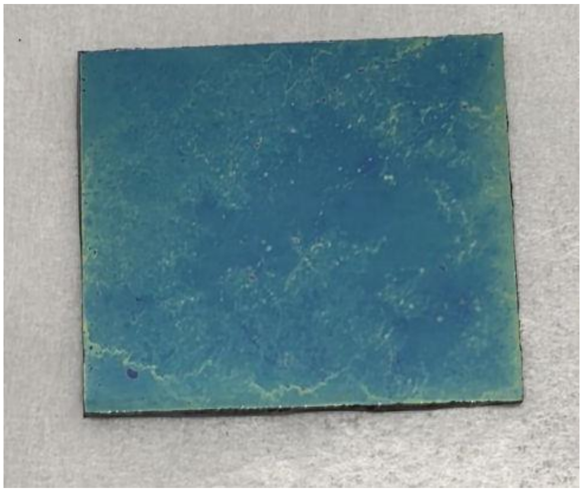

Figure S15. Photo of TAPB-DHPDA COF on SiO_2/Si substrate

GIWAXS Data Analysis

	COF	Peak	Position	FWHM	Amplitude	Avg. Position	Avg. FWHM	Avg. Amplitude
Mixed Orientation	**TAPA-TFB (Before)**	Peak 1	0.47	0.04	397	**0.47**	**0.03**	**393**
		Peak 2	0.46	0.03	390			
	**TAPA-TFB (After)**	Peak 1	0.47	0.04	1391	**0.47**	**0.04**	**1397**
		Peak 2	0.46	0.03	1404			
	**TAPB-TFB (Before)**	Peak 1	0.39	0.04	945	**0.39**	**0.04**	**937**
		Peak 2	0.39	0.04	929			
	**TAPB-TFB (After)**	Peak 1	0.40	0.04	784	**0.39**	**0.04**	**780**
		Peak 2	0.39	0.04	776			
	**TAPB-OHTFB (Before)**	Peak 1	0.41	0.04	1804	**0.41**	**0.04**	**1804**
		Peak 2	0.40	0.04	1804			
	**TAPB-OHTFB (After)**	Peak 1	0.41	0.04	817	**0.40**	**0.04**	**803**
		Peak 2	0.40	0.04	790			
Parallel Orientation	**TAPB-DHPDA (Before)**	Peak 1	0.20	0.06	1269	**0.20**	**0.06**	**1281**
		Peak 2	0.20	0.06	1292			
	**TAPB-DHPDA (After)**	Peak 1	0.20	0.04	7780	**0.20**	**0.04**	**7813**
		Peak 2	0.20	0.04	7846			
	**DA-OHTFB (Before)**	Peak 1	0.33	0.03	286509	**0.33**	**0.02**	**292509**
		Peak 2	0.33	0.02	298509			
	**DA-OHTFB (After)**	Peak 1	0.34	0.02	142161	**0.33**	**0.02**	**146277**
		Peak 2	0.33	0.02	150393			
	**TAPB-PDA (Before)**	Peak 1	0.19	0.05	183	**0.19**	**0.05**	**187**
		Peak 2	0.19	0.05	191			

	**TAPB-PDA (After)**	Peak 1	0.20	0.02	1872	**0.20**	**0.02**	**1892**
		Peak 2	0.20	0.02	1912			

Table S1. GIWAXS Data Analysis

Determination of COF Pore Size from GIWAXS

The COF structures described in this manuscript have an analogous structure to that of "COF-5" as originally described by Côté et al[1], namely that formed by an eclipsed or nearly eclipsed[2] stacking of hexagonally-symmetric, honeycomb-like sheets. The conventional unit cell for these structures forms a rhombus in the plane of these sheets as shown in **Figure S16**, with in-plane basis vectors **a** and **b** 120° apart.

For many potential applications, a critical design parameter for COFs is the effective pore size. In general, this size is determined by the dimensions of the unit cell, the diameter of the molecular chains forming the structure, and the degree of alignment between layers, and is often estimated from gas adsorption and modeling. In this work, we adopt the definition of the pore size, as the length of the in-plane hexagonal basis vector |**a**| = |**b**|, which corresponds to the distance between the centers of the edges forming each polygonal section of the COF. From inspection of **Figure S16**, this length is equal to $2/\sqrt{3}$ times the d-spacing of the (100) Bragg Peak, which is the first and most prominent peak measured in the GIWAXS measurements obtained for this study. The values used in the manuscript to characterize the pore size of the 6 different COF systems discussed are therefore equal to the hexagonal lattice parameter measured from GIWAXS (see **Figures 2-6** as well as **Figure S2**, **Figure S3**, and **Figure S14**). We note that the physical pore size, corresponding to the inner diameter of each pore, is necessarily smaller than calculated pore size.

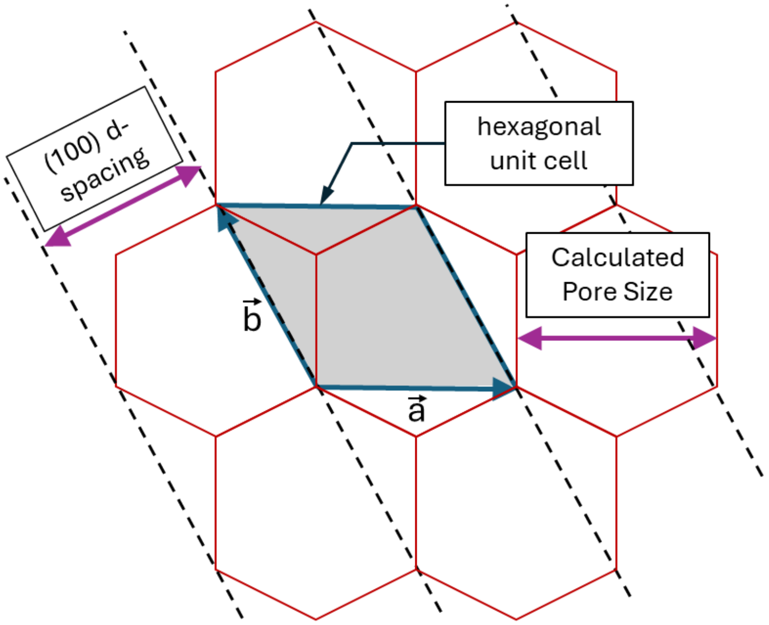

Figure S16. Definition of pore size in hexagonal COFs.

	COF	% Change Position	% Change FWHM	% Change Amplitude	Å^{-1} of (100) Peak	(100) d_{Bragg} (nm)	Pore $Size_{XRD}$ (nm)
Mixed Orientation	**TAPA-TFB**	0%	1%	255%	0.47	**1.35**	**1.56**
	**TAPB-TFB**	1%	-10%	-17%	0.39	**1.60**	**1.85**
	**TAPB-OHTFB**	0%	14%	-55%	0.40	**1.56**	**1.80**
Parallel Orientation	**TAPB-DHPDA**	0%	-37%	510%	0.20	**3.15**	**3.64**
	**DA-OHTFB**	1%	-8%	-50%	0.33	**1.88**	**2.17**
	**TAPB-PDA**	5%	-61%	911%	0.20	**3.13**	**3.61**

Table S2. Calculated Percent Change and d-spacing

Determination of the Critical Angle

Sample Alignment

Sample alignment was performed with the aid of a diode or ion chamber placed downstream of the sample. Importantly, either the finite size of the diode or a slit blade placed on the ion chamber immediately above the direct beam were used to prevent detection of reflected intensity from the sample surface. With any reflected intensity thus blocked, a scan of the incident angle of a sample that partially blocks the beam will have maximum intensity when the sample is parallel to the beam.

The sample is aligned by iteratively adjusting or scanning the sample height and incident angle until the sample blocks about 50% of the incident beam and the sample surface is parallel to the beam. These two conditions are verified by final scans of the sample height and incident angle in succession. Once met, the "theta" angle is assigned to 0, and the sample height is maintained at that height.

Measurement of θ-Dependent Scattering and Extraction of θ_c

Following alignment, the incident angle was scanned from 0.03° to 0.20°. At each θ value, the scattering intensity was integrated over a fixed region of the detector corresponding to the primary COF diffraction feature (**Figure S17**).

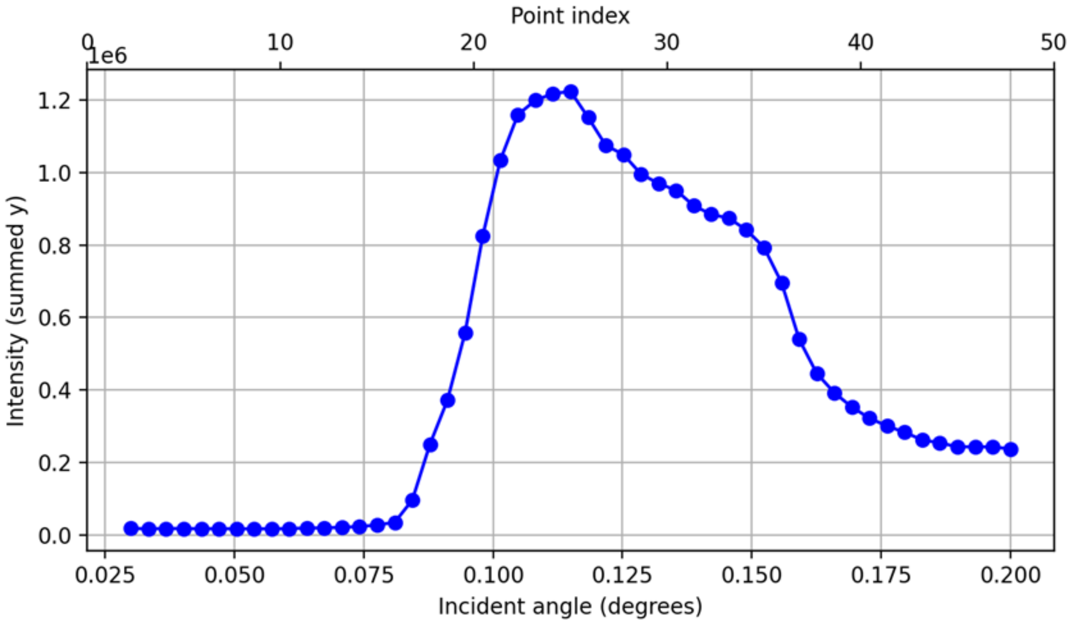

Figure S17. Graph of Scattering Intensity Determined from Incident Angle (°)

As θ increases, the illuminated volume and evanescent-wave penetration depth increase, leading to a corresponding rise in the scattered intensity. The critical angle, θ_c, was defined as the incident angle at which this integrated scattering intensity reached its maximum. Across samples, θ_c typically ranged from 0.11° to 0.15°, reflecting slight variations in film thickness and density.

Beam Footprint Analysis

Beam Dimensions and Footprint Geometry

The incident X-ray beam had a size of approximately 45 μm (vertical) × 500 μm (horizontal). At grazing incidence, the vertical beam height projects into an elongated footprint on the sample surface. The footprint length L is given by:

$$L = \frac{h}{\sin\theta}$$

where h is the vertical beam size.

Representative footprint lengths are listed in **Table S3**.

**Incident Angle θ**	**sin θ**	**Footprint Length** $L = 45\ \mu m / \sin\theta$
0.10°	0.00175	~25.9 mm
0.12°	0.00209	~21.5 mm
0.15°	0.00262	~17.2 mm

Table S3. Footprint Length Calculation

Given that the COF samples were approximately 20–30 mm long, these calculations show that the beam footprint was comparable to the sample length throughout the θ-scan. As a result, precise sample height and angle alignment were essential to ensure the entire beam footprint remained on the sample.

Impact on Critical Angle Measurement

Because the footprint often approached the total sample length, small deviations in sample height or tilt could lead to partial illumination, geometric clipping, or intensity loss unrelated to material properties. Ensuring full footprint coverage during the θ-scan minimized these geometric effects, allowing the measured intensity maximum to provide a consistent determination of the critical angle.

References

(1) Côté, A. P.; Benin, A. I.; Ockwig, N. W.; O'Keeffe, M.; Matzger, A. J.; Yaghi, O. M. Porous, Crystalline, Covalent Organic Frameworks. *Science* **2005**, *310* (5751), 1166–1170. https://doi.org/10.1126/science.1120411.

(2) Bisbey, R. P.; Dichtel, W. R. Covalent Organic Frameworks as a Platform for Multidimensional Polymerization. *ACS Cent. Sci.* **2017**, *3* (6), 533–543. https://doi.org/10.1021/acscentsci.7b00127.
